# Dynamic Expression Changes in the Transcriptome of the Prefrontal Cortex after Repeated Exposure to Cocaine in Mice

**DOI:** 10.3389/fphar.2017.00142

**Published:** 2017-03-23

**Authors:** Mingzhen Li, Peng Xu, Yanhua Xu, Huajing Teng, Weiping Tian, Quansheng Du, Mei Zhao

**Affiliations:** ^1^Key Lab of Mental Health, Institute of Psychology Chinese Academy of SciencesBeijing, China; ^2^Beijing Center for Physical and Chemical AnalysisBeijing, China; ^3^Drug Intelligence and Forensic Center, Ministry of Public SecurityBeijing, China; ^4^Beijing Institutes of Life Science, Chinese Academy of SciencesBeijing, China; ^5^University of Chinese Academy of SciencesBeijing, China; ^6^Department of Life Sciences, National Natural Science Foundation of ChinaBeijing, China

**Keywords:** prefrontal cortex (PFC), transcriptome, cocaine, addiction, withdrawal

## Abstract

Prefrontal cortex (PFC)-dependent functions, such as executive function, explicit learning, and memory, are negatively affected in cocaine abusers and experimental animal models of cocaine treatment. However, its molecular mechanisms are less understood. In the present study, we performed transcriptome profiling of the dynamic changes in the PFC after repeated cocaine administration in mice. We found 463, 14, and 535 differentially expressed genes (DEGs) at 2 h, 24 h, and 7 days, respectively, after the withdrawal of chronic cocaine treatment. Time-series correlation analysis identified 5 clusters of statistically significant expression patterns. The expression levels of DEGs in Clusters 1 and 5 exhibited a gradual or fluctuant decrease, Cluster 2 exhibited an initial increase followed by a decrease or return to the baseline level, and Clusters 3 and 4 exhibited a fluctuant increase in the expression of DEGs. The Kyoto Encyclopedia of Genes and Genomes (KEGG) pathway analysis revealed that genes related to oxidative phosphorylation, ribosomes, and neurodegenerative disorder were enriched in Cluster 1; genes related to the mitogen activated protein kinase (MAPK), transforming growth factor (TGF)-β, insulin signaling, and circadian pathways were enriched in Cluster 2; genes related to plasticity-related pathways were enriched in Clusters 3 and 4; and genes related to the proteasome were enriched in Cluster 5. Our results suggest that maladaptive neural plasticity associated with psychostimulant dependence may be an ongoing degenerative process with dynamic changes in the gene network at different stages of withdrawal. Furthermore, it could be helpful to develop new therapeutic approaches according to different periods of abstinence.

## Introduction

Drug addiction is a chronic relapsing brain disease that is characterized by compulsive out-of-control drug use, despite serious negative consequences (Pol bodetto et al., 2013). This behavioral abnormality is caused by repeated exposure to a drug of abuse, and can potentially persist life-long (Nestler, 2004). This persistence suggests that drug-induced maladaptive neural plasticity drives these long-term behavioral abnormalities, and changes of gene expression in brain reward regions play important roles in this process (Chao and Nestler, 2004; Nestler, 2004; Hyman et al., 2006).

The mesolimbic dopamine system is generally considered the most important mediator of drug reward, and cocaine has a distinct effect on different anatomical regions in the brain. Dopamine neurons are located in the ventral tegmental area (VTA) of the midbrain and project to the limbic forebrain, including the nucleus accumbens (NAc) and prefrontal cortex (PFC), while the PFC sends glutamatergic efferents to the VTA and NAc (Chao and Nestler, 2004). Cocaine has distinct effects on these regions. The VTA-NAc circuit is the most intensively studied region as it is thought to play an important role in the initial rewarding effects of cocaine (Chao and Nestler, 2004), while the roles of the PFC, an area critical for cognition and executive functions, are less understood. It is suggested that the PFC contributes to both the primary reinforcing effects of cocaine and long-term sensitization to cocaine (Chao et al., 2014). PFC-dependent functions, such as executive function, explicit learning, and memory were negatively affected in cocaine abusers and experimental animal models of cocaine treatment (Chao et al., 2014).

Over the past decade, many genes including various membrane receptors, signal transduction molecules, and nuclear genes, have been identified that drive addiction-related behaviors (Palanza, 2001; Itzhak et al., 2014). However, most drug-induced gene expression changes are very transient and do not fully explain the long-lived alterations in addiction-related behavior (Sen, 2014). It is suggested that the expression or transient-differential-expression of many genes such as immediate-early genes and transcription factors may affect the expression of other genes, and it is of particular importance to investigate gene expression long-after exposure to addictive drugs (Jacobs et al., 2005). In addition, change in the expression of most genes induced by exposure to addictive drugs might have a specific spatiotemporal profile. In this case, gene networks rather than individual genes are thought to be altered, and therefore lead to more enduring neuro-adaptations that drive structural plasticity to control additional behaviors (Palanza, 2001; Chao et al., 2014; Itzhak et al., 2014). Whole-genome gene expression profiles may help reveal the complex gene-gene interaction mechanisms of addiction. Thus, in the current study, we performed transcriptome profiling of the mouse PFC to determine the dynamic changes in this region after repeated cocaine treatment, to understand long-lasting changes in the gene network after cocaine administration.

## Materials and methods

### Cocaine treatment and PFC dissection

Adult male (10-week-old) C57BL/6 J mice (purchased from HFK Bioscience Co., Ltd, Beijing, China) were used in this study. Animals were habituated for at least 1 week in the new environment before experimentation. They were housed 5 per cage, on a 12 h light-dark cycle, and in a constant temperature environment (21 ± 1°C), with access to food and water *ad libitum*.

All mice were randomly divided into cages, with five mice per cage. Cocaine was dissolved in 0.9% saline. Repeated-cocaine treatments is a popular paradigm to explore the mechanisms of cocaine addiction. Consecutive intraperitoneal injections for 7 or 14 days are commonly used in previous studies, which had been demonstrated neuroadaptational changes associated with cocaine addition (Feng et al., 2014; Puig et al., 2014). In the present study, animals received intraperitoneal injections of cocaine (Qinghai Pharmacoceutical, China) at a dose of 20 mg/kg/d during 9:00 to 11:00 a.m. for 14 consecutive days. All control groups received saline injections for 14 consecutive days. Mice were asphyxiated using dry ice, and then the bilateral PFC (0–1.5 mm from the tip of brain) was excised from each animal individually at 2 h, 24 h, or 7 days after the final injection of cocaine and then pooled randomly. The cortical subregions in the excised tissue included frontal association cortex, prelimbic cortex and orbital cortex. To account for inter-animal variations, we obtained 2 biological replicates for each treatment group, with each replicate representing the PFCs pooled from 5 animals. The tissue was stored in liquid nitrogen immediately and then transferred to −80°C refrigerator. All animal protocols were approved by the Review Board of the Institute of Psychology, Chinese Academy of Sciences and were performed strictly in accordance with the Guideline for Care and Use of Laboratory Animals of the Chinese Academy of Sciences.

### RNA sequencing and functional enrichment analysis

To characterize the transcriptome of the PFC, we used RNA sequencing (RNA-seq) to measure the expression levels of all polyA-containing transcripts in the PFC of mice treated chronically with cocaine or saline (control).

RNA was extracted from PFC tissues using Trizol (Invitrogen, Cat. 15596026) following the manufacturer's instructions. Briefly, 50–100 mg tissue was homogenized in 1 ml of TRIzol using a homogenizer. RNA was separated by adding 0.2 ml of chloroform per 1 ml of TRIzol reagent and then precipitated with isopropanol. RNA quantified using a NanoDrop 2000 (Thermo-Fisher Scientific, Waltham, MA, USA), and assessed with an Agilent 2100 Bioanalyzer (Agilent, Santa Clara, CA, USA). The RNA integrity number (RIN) of each sample was >8.

Total RNA (240–380 ng/ul, 2 ug) was used to prepare cDNA libraries according to the manufacturer's protocol (TruSeq™ RNA Sample Prep Kit v2 -Set A, Illumina, San Diego, CA, USA). The brief procedure of library preparation is as follows: enrichment of mRNA by poly A, mRNA fragmentation, first strand synthesis, second strand synthesis, end repair, A tailing, adaptor ligation and PCR (13 cycle, 22.4–26.6 ng/ul × 30 ul).

Paired-end 100 nt sequencing was performed using the Illumina HiSeq2000 (TruSeq PE Cluster Kit v3–cBot - HS and TruSeq SBS Kit v3–HS). These 12 raw sequencing data sets were deposited in the Gene Expression Omnibus of the NCBI (https://www.ncbi.nlm.nih.gov/geo) under accession number GSE89572.

### Bioinformatic processing of the RNA-seq data

After removal of the adaptor sequences and trimming of low quality sequences using cutadapt (Martin, 2011), all reads were aligned to the UCSC mm9 version of the mouse genome assembly using the TopHat2 short read alignment program(Trapnell et al., 2009). Only uniquely mapped reads were used in this analysis. Fragments Per Kilobase of sequence per Million mapped reads (FPKM) were used to normalize the number of aligned reads by the size of the gene and the total number of mapped reads. The changes in gene expression between different groups were analyzed using the Cufflinks 2.0 package (Trapnell et al., 2012). Cufflinks can estimate the relative abundances of these transcripts based on how many reads support each one, taking into account biases in library preparation protocols (http://cole-trapnell-lab.github.io/cufflinks/manual/). Difference in expression of genes with FDR (False Discovery Rate) value < 0.05 were considered as differentially expressed genes.

We identified statistically enriched functional terms from the public databases of the KEGG Pathways (http://www.genome.jp/kegg/pathway.html). Functional terms with FDR-value < 0.05 was considered as statistically significant enrichment.

Time-series clustering was performed to identify statistically significant expression patterns and gene groups among differentially expressed genes (DEGs) using the short time-series expression miner (STEM; Ernst and Bar-Joseph, 2006). STEM uses a method of analysis that takes advantage of the number of genes being large and the number of time points being few to identify statistically significant temporal expression profiles and the genes associated with these profiles. User-defined parameters were set to 35 profiles, maximizing the number of clustering genes, with a minimum correlation threshold of 0.7. Five statistically significant clusters were identified (*p* < 0.05).

### Validation of differentially expressed genes (DEGs)

cDNA was synthesized from 1 μg of total RNA (template) using Superscript III (Invitrogen, CA, USA). The validation of DEGs were determined by real-time quantitative PCR using SYBR Green Mix (CWBIO, Beijing, China). PCR conditions were: 95°C for 10 min, 40 cycles at 95°C for 10 s, 60°C for 60 s, and signal detection for 10 s; dissociation curve analysis was performed at the end of each run, and relative mRNA expression was determined by the standard ΔΔCt method using glyceraldehyde-3-phosphate dehydrogenase (GAPDH) as a normalization control. The primers are listed in Table S1.

### Data analysis

Statistical analyses were performed with GraphPad Prism 6.0 software. Means of relative gene expression between two groups were compared with a two-tailed Student's *t*-test. Statistical significance was set at *p* < 0.05.

## Results

### Quality assessment of the sequenced data

We obtained 36, 913, 048 to 50, 325, 962 short reads of 100 bp from each replicate from both the cocaine treated and control animals. Of these, 84–87.08% were successfully aligned to a reference gene database (Ensembl: *Mus musculus*, NCBI) by TopHat (Trapnell et al., 2009).

### Overview of the DEGs

**Figure 1:**
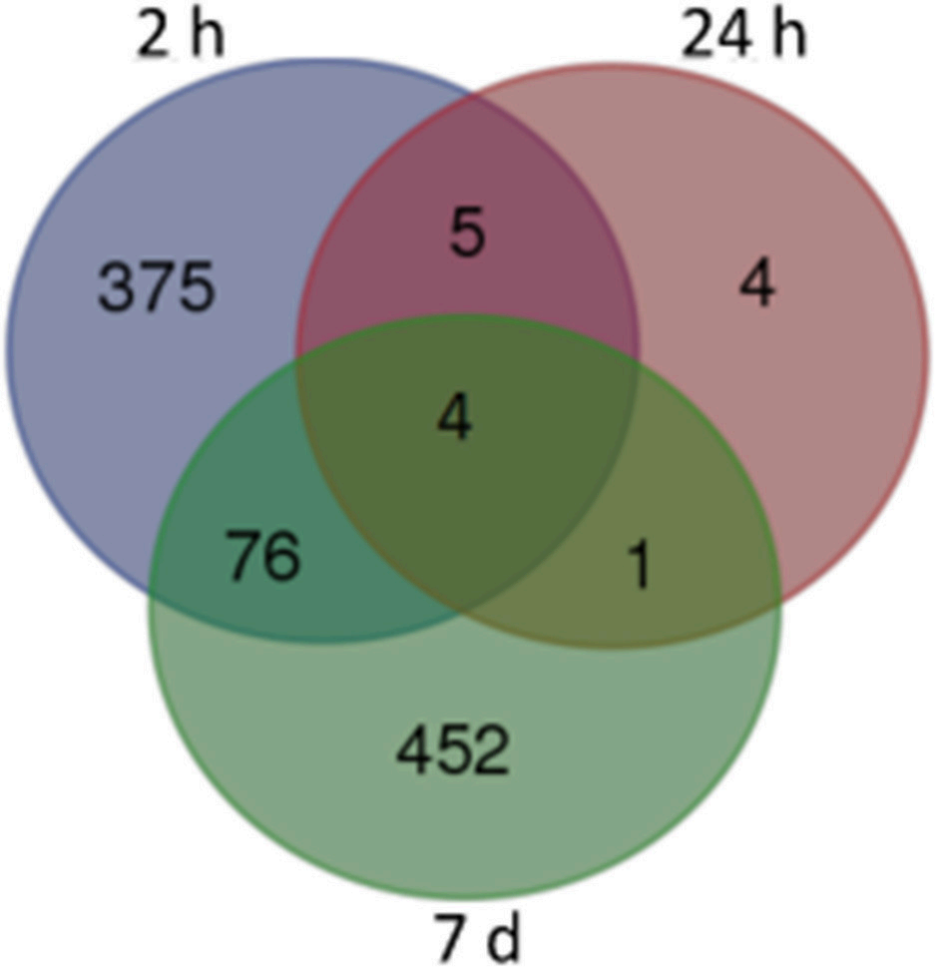
**Venn map of DEGs from different withdrawal time: subsets of significant changes by Tukey's HSD comparison**. The numbers within each segment indicate the number of DEGs of different withdrawal time points.

For our initial analysis, we used stringent **false discovery rate** (FDR) cutoffs of < 0.05. As showed in Figure 1, we obtained 463, 14, and 535 DEGs from samples obtained 2 h, 24 h, and 7 days after the final administration of cocaine, respectively (corrected *p* < 0.05). Among of them, there were 62/401 (2 h), 3/11 (24 h), and 311/224 (7 d) DEGs with decreased/increased expression level, respectively (Tables S2, S3 and S4). Only 4 DEGs were shared by all 3 time points, namely *Dusp6, Fos, Nr4a1*, and *Egr1* (Figure 1).

Through functional enrichments of DEGs, several signaling pathways were enriched at each of the time points (Table 1). Two categories of signaling pathways were enriched (corrected *p* < 0.05) at 2 h. The first category comprised the signaling pathways regulating cell division, cell differentiation, and cell death, e.g., the Wnt signaling pathway, mitogen activated protein kinase (MAPK) signaling pathway, and Jak-STAT signaling pathway. The second enriched pathway was related to the circadian rhythm. Even though the number of DEGs was greatly decreased after 24 h of withdrawal, the circadian rhythm and MAPK signaling pathway were significantly enriched. At the 7 days post-withdrawal time-point, the number of DEGs was increased; however, most of these genes were significantly enriched (FDR < 0.01) in metabolic pathways, for example, the electron transport chain, ribosomes, proteasomes, citrate cycle, and RNA polymerase. These results indicate that distinct pathways are involved in the acute, sub-acute, and long-lasting stages of withdrawal after chronic cocaine treatment.

**Table 1:** **Distinct pathways involved in different stages of withdrawal after chronic cocaine treatment**.

**2 h after final cocaine administration**			
**#**	**Pathway**	**FDR**	**Pathway ID**
1	Pathways in cancer	0.005854	ko05200
2	Circadian rhythm - mammal	0.005854	ko04710
3	Adherens junction	0.006437	ko04520
4	Circadian rhythm - fly	0.011379	ko04711
5	Jak-STAT signaling pathway	0.011575	ko04630
6	Melanogenesis	0.029236	ko04916
7	Wnt signaling pathway	0.029236	ko04310
8	MAPK signaling pathway	0.029236	ko04010
**24 h after final cocaine administration**			
#	Pathway	FDR	Pathway ID
1	Circadian rhythm - mammal	0.00279	ko04710
2	MAPK signaling pathway	0.013751	ko04010
3	Circadian rhythm - fly	0.030747	ko04711
**7 days after final cocaine administration**			
#	Pathway	FDR	Pathway ID
1	Oxidative phosphorylation	4.86E-32	ko00190
2	Ribosome	3.71E-31	ko03010
3	Parkinson's disease	5.02E-27	ko05012
4	Huntington's disease	1.70E-26	ko05016
5	Alzheimer's disease	2.96E-24	ko05010
6	Proteasome	2.00E-07	ko03050
7	Cardiac muscle contraction	3.95E-06	ko04260
8	Vibrio cholerae infection	2.19E-05	ko05110
9	Metabolic pathways	2.63E-05	ko01100
10	Rheumatoid arthritis	0.001783	ko05323
11	Epithelial cell signaling in Helicobacter pylori infection	0.001876	ko05120
12	Collecting duct acid secretion	0.004512	ko04966
13	RNA polymerase	0.007616	ko03020
14	Citrate cycle (TCA cycle)	0.008566	ko00020
15	Protein export	0.009591	ko03060
16	Gastric acid secretion	0.011006	ko04971
17	Salivary secretion	0.015994	ko04970
18	Vascular smooth muscle contraction	0.016231	ko04270
19	Aldosterone-regulated sodium reabsorption	0.028328	ko04960
20	Long-term potentiation	0.035467	ko04720

*FDR mean “false discovery rate.”

### Clusters from stem analysis and their KEGG pathway analysis

**Figure 2:**
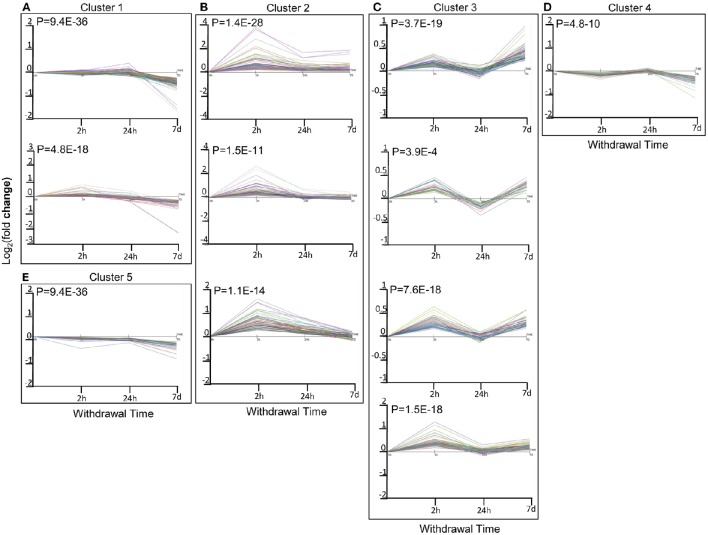
**Correlated expression Clusters and their sub-clusters discovered using the short time series expression miner (STEM) algorithm for time series profile matching: (A)**: Cluster 1 and its sub-cluster; **(B)**: Cluster 2 and its sub-cluster; **(C)**: Cluster 3 and its sub-cluster; **(D)**: Cluster 4 and its sub-cluster; **(E)**: Cluster 5 and its sub-cluster. Different colors within each sub-cluster represented different genes. Values are the mean Log_2_(fold change) values as compared to sacrifice-time matched control animals.

To further utilize the dynamic expression information, we performed time-series correlation analysis on the total DEGs of all 3 time points using the STEM method, which produced 5 clusters of DEGs with significant serial correlation of expression changes (*r* > 0.7; *p* < 0.05). Among them, the expression levels of DEGs in Cluster 1 gradually decreased with time, while those in Cluster 2 first increased and then decreased or returned to the baseline level (the same level as those in the saline group). Expression changes in Cluster 3 and Cluster 4 shared a similar pattern of fluctuation, with an initial increase followed by a decrease and then finally another increase. Whereas, expression levels of DEGs in Cluster 5 initially decreased, then increased, and decreased again at the end (Figure 2).

### Clusters 1 and 5: gradual or fluctuant decrease

**Figure 3:**
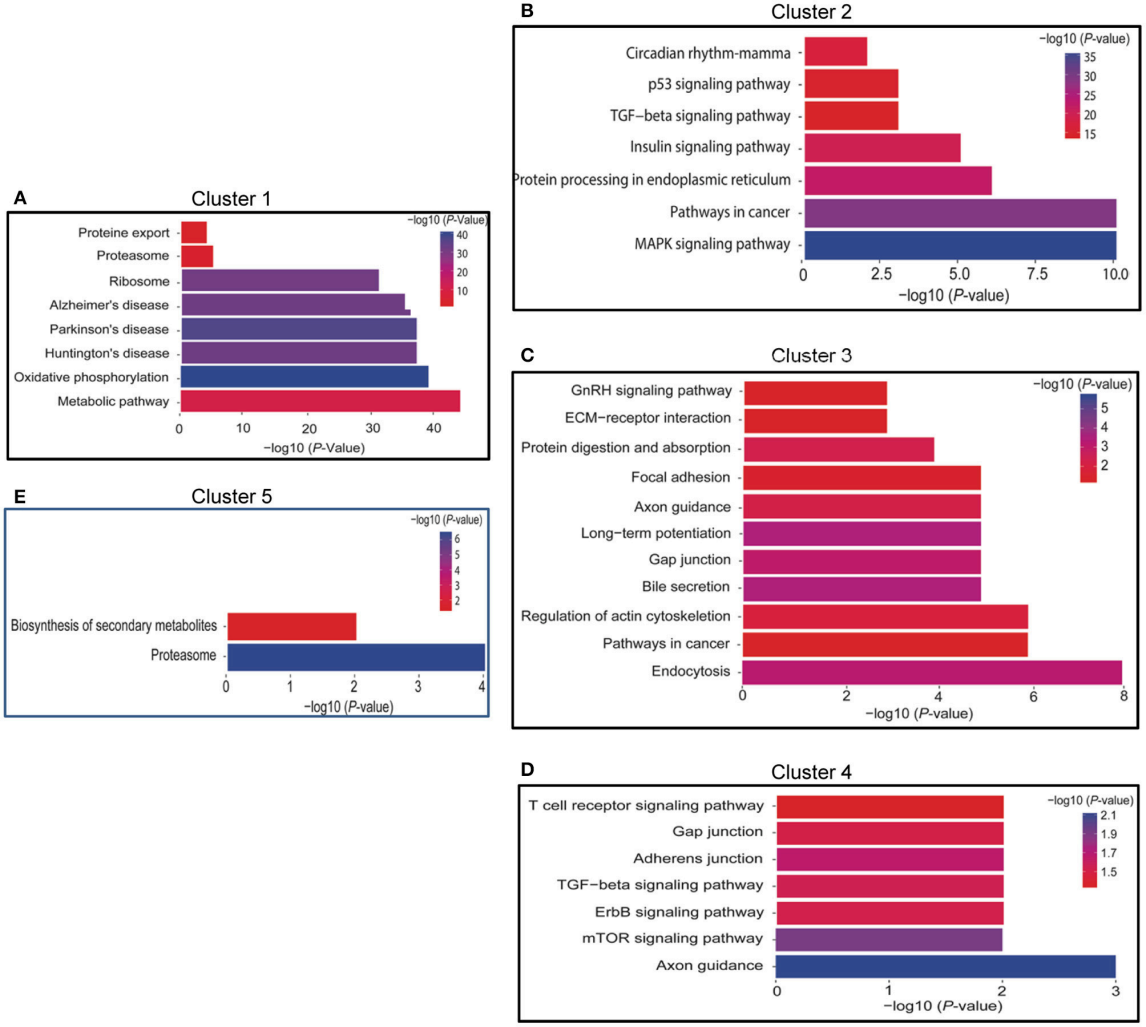
**Functional annotation of DEGs of Time series clusters by KEGG pathways. (A)** Functional annotation of DEGs of cluster 1; **(B)**: Functional annotation of DEGs of cluster 2; **(C)**: Functional annotation of DEGs of cluster 3; **(D)**: Functional annotation of DEGs of cluster 4; **(E)**: Functional annotation of DEGs of cluster 5. The length of the bar represent the number of gene involved in the pathway.

There were 3 sub-clusters in Cluster 1 that were consistent with the general tendency of gradual or fluctuant decrease, as shown in Figures 2A, 3A. The most highly enriched pathways in Cluster 1 were oxidative phosphorylation, and neurodegenerative disorders such as Huntington's disease, Parkinson's disease, and Alzheimer's disease. In addition, the oxidative phosphorylation chain and proteasome were significantly enriched in both Clusters 1 and 5 (Figures 2E, 3E).

### Cluster 2: initial increase followed by a decrease or return to the baseline

There were 3 sub-clusters in Cluster 2 as showed in Figures 2B, 3B. KEGG pathway analysis of genes within Cluster 2 showed that the MAPK signaling pathway, the p53 pathway, TGF-β signaling pathway, insulin-signaling pathway, and circadian pathway were significantly enriched.

### Clusters 3 and 4: fluctuant increase

Clusters 3 and 4 (Figures 2C,D, 3C,D) shared a similar pattern of changes in the expression of DEGs, an initial increase followed by a decrease and then finally an increase. KEGG pathway analysis of Clusters 3 showed that they were mainly enriched in plasticity-related pathways, such as long-term potentiation and the regulation of the actin cytoskeleton, while axon guidance was enriched in both Clusters 3 and 4.

### Validation of RNA-seq data by qRT-PCR

We validated these RNA-seq data independently from tissue samples using qRT-PCR. As shown in Table 2, we identified the expression of 10 genes at 3 different withdrawal time points, with 83.3% of genes/time points showing consistent results. Some of validated genes have been widely studied on their effects on cocaine exposure, while others have known functions, which may contribute to drug addiction. *Fosb* encoded ΔFosB, a truncated and stable protein, which is a well-characterized transcription factor induced by chronic exposure to virtually all drugs of abuse (Wallace et al., 2008). Nr4a1 increases in frontal cortex (Freeman et al., 2002) and Nr4a3 increases in the medial caudate putamen and cingulate cortex (Werme et al., 2000) after chronic cocaine administration. *Nr4a2* plays an important role in development and differentiation of midbrain DA neurons and *Nr4a2* gene expression decreased in human post-mortem midbrain of cocaine abusers (Leo et al., 2007). Our results further demonstrated that Nr4a1 persistently increased while Nr4a2 and Nr4a3 showed transient changes after cocaine withdrawal, indicating their different roles in different stages of cocaine addiction. The roles of Wnt pathway in the NAc in long-term cocaine-induced neuroplasticity have been reported (Cuesta et al., 2017). Notch signaling is an important regulator of neuronal homeostasis and Notch1 activation is involved in cocaine-mediated regulation of PDGF-B expression (Yao et al., 2011). In the present study, we found both *Wnt4* and *Notch2* expression increased 2 h after withdrawal and their roles in cocaine addiction should be further investigated. Cocaine-induced histone methylation at many specific candidate genes is already known (11). We found Kdm6b, a histone demethylase, increased 7d after cocaine withdrawal, indicating its potential role in cocaine addiction for the first time.

**Table 2:** **Comparison of RNA-seq and RT-PCR**.

**Gene**	**RNA-Seq**			**RT-PCR**		
	**2 h**	**24 h**	**7 d**	**2 h**	**24 h**	**7 d**
*JunB*	5.4^*^	1.67	1.38^*^	3.70^*^	1.37	1.66^*^
*Nr4a1*	4.53^*^	1.62^*^	1.51^*^	4.37^*^	2.36^*^	1.29^*^
*Nr4a2*	2.03^*^	1.7	1.32	1.40^*^	1.26	1.36
*Nr4a3*	3.01^*^	1.69	1.18	2.10^*^	1.14	0.99
*Kdm6b*	1.00	1.17	1.18	1.16	1.17	1.31^*^
*Notch1*	1.36	1.07	1.22	1.01	0.87	1.18
*Notch2*	1.43^*^	0.92	1.33	0.88	0.81	1.01
*Wnt4*	1.33^*^	1.23	1.14	1.08	1.07	0.84
*Wnt7b*	1.03	1.004	1.005	0.69^*^	0.95	0.73
*Grin2b*	1.09	0.96	1.38^*^	0.92	0.83	1.25

* *p < 0.05 compared with saline control*.

## Discussion

In the current study, we observed dynamic changes in the transcriptome profiling of the PFC of repeated-cocaine treated mice, and found that distinct pathways were involved in the acute, sub-acute, and chronic stages of withdrawal. The main findings of our results include: (1) energy metabolism and protein metabolism pathways showed gradual or fluctuant decrease after cocaine withdrawal; (2) ERK pathway showed persistent changes after cocaine withdrawal; (3) plasticity related pathways, such as long-term potentiation, the regulation of the actin cytoskeleton, and the axon guidance pathway, showed a fluctuant increase after cocaine withdrawal.

### Dynamic decrease of energy metabolism and protein metabolism pathways

Mitochondrial energy production, which occurs through oxidative phosphorylation, requires the action of various respiratory complexes together known as the electron transport chain enzymes (ETC). There are 5 ETC complexes: (i) Complex I (NADH dehydrogenase), (ii) Complex II (succinate dehydrogenase), (iii) Complex III (cytochrome bc1 complex), (iv) Complex IV [cytochrome c oxidase (COX)], and (v) Complex V (ATP synthase). Our results found that over 40 genes in the PFC involved in each ETC complex were gradual or fluctuant decrease after cocaine-withdrawal, indicating that energy metabolism impairment induced by repeated-cocaine treatment showed a time-dependent increase following withdrawal. Studies in humans, nonhuman primates, and mice have demonstrated cocaine-associated reductions in metabolic activity in the brain. By testing the regional cerebral metabolic rate for glucose, it was found that 10 or 14 days of repeated daily treatment decreased metabolic activity in the mesolimbic dopaminergic pathway, including the PFC (Hammer and Cooke, 1994). The glucose metabolism rate and blood flow significantly decreased in the PFC of cocaine addicts, indicating lower metabolism and activity in brain of cocaine abusers (Goldstein and Volkow, 2002; Volkow et al., 2004). At the molecular level, both metabolic proteins in cocaine addicts and mitochondrial proteins in animal model of cocaine abuse are decreased (Lull et al., 2008). Our results further showed a dynamic metabolic decrease after cocaine withdrawal and suggest that this gradual decrease could represent an adaption of the metabolic response to cocaine in the PFC. A recent study found that compulsive drug-seeking behavior induced by repeated cocaine treatment resulted from reduced pyramidal cell excitability mediated by PFC metabolic dysfunction (Chen et al., 2013). Here, we revealed changes of the energy metabolism network underlying repeated cocaine treatment and believe that an adaption of the metabolic response to cocaine in the PFC may be another important mechanism of relapse during protracted abstinence.

Dynamically decreased genes were also enriched in the proteasome pathway, including the subunits composing the core particle of proteasomes during ubiquitin-mediated proteolysis. The ubiquitin proteasome system (UPS) controls the degradation of misfolded, newly synthesized proteins, as well as the turnover of specific target proteins. Consistent with our results, many transcriptomic and proteomic studies have described the effects of treatment with drugs of abuse on proteasome subunits or proteins involved in the ubiquitination process (Massaly et al., 2014). In the case of cocaine treatment, ubiquitin-conjugating enzyme E2N and proteasome subunit-alpha type 2 were involved in cocaine-conditioned place preference (Guan and Guan, 2013). Interestingly, it is suggested that synaptic and signaling proteins, especially transcription factors, which mediate enduring neuronal plasticity, could be regulated directly or indirectly by **a UPS-dependent process** (Massaly et al., 2014). Combined with lasting changes in transcription factors such as *Nr4a1, Fos*, and *Egr1* in our results, we speculate that UPS-dependent regulation plays an important role in neuronal plasticity associated with the long-term effect of cocaine.

### ERK pathway showed persistent changes after cocaine withdrawal

The MAPK pathway is one of the most conserved signaling pathways in eukaryotes. Multiple proteins involved in this pathway transfer extracellular biological, chemical, or physical signals into the cell by adding or removing adjacent phosphate groups of the protein molecule(Roux and Blenis, 2004). The MAPK signal pathway is divided into 3 sub-pathways: the ERK (extracellular signal-regulated kinase), JNK (c-Jun NH_2_-terminal kinase), and p38 signal pathways (Roux and Blenis, 2004). **Four** genes, phosphorylase Dusp6, and transcription factors Egr1, Fos, and Nr4a1, which all belong to the ERK signaling pathway, showed a persistent expression change after cocaine withdrawal (Table 3).

**Table 3:** **DEGs involved in the MAPK signal pathway**.

**Withdrawal time**	**Pathway**	**ERK**	**p38**	**JNK**
2 h	A.S./Receptor	*Pdgfb/Cacnb1*	–	–
	MAPKKK	–	*Gadd45*	–
	MAPKK	–	–	Crk1
	MAPK/MAPK Phosphatase	Dusp1, Dusp4, Dusp5, Dusp6	p38/Dusp1, Dusp4, Dusp5, Dusp6	Dusp1, Dusp4, Dusp5, Dusp6
	Downstream effectors	Mknk2, Rps6ka2, Fos, Nr4a1	Hsp27	–
24 h	A.S./Receptor	–	–	–
	MAPKKK	–	–	–
	MAPKK	–	–	–
	MAPK/MAPK Phosphatase	Dusp6	Dusp6	Dusp6
	Downstream effectors	Fos, Nr4a1	–	–
7 d	A.S./Receptor	Fgfr3	–	–
	MAPKKK	Prkcb (PKC)	–	–
	MAPKK	–	–	–
	MAPK/MAPK Phosphatase	Dusp1, Dusp6	Dusp1, Dusp6	Dusp1, Dusp6
	Downstream effectors	Stmn1, Fos, Nr4a1	–	–

Several lines of evidence have demonstrated the important roles of the ERK pathway in cocaine addiction. Both acute and repeated injections of cocaine activated ERK in the mesolimbic reward system, including the VTA, PFC, bed nucleus of the stria terminalis, and amygdala (Lu et al., 2006). In addition, ERK contributes to various behavioral effects of cocaine, such as psychomotor sensitization, conditioned place preference, and reconsolidation of memories for cocaine cues (Lu et al., 2006). Importantly, long-lasting changes of the ERK pathway induced by repeated cocaine treatment have been supported by the association of long-term ERK-mediated effects and ERK-dependent activation of FOS and EGR1 (Radwanska et al., 2005). High rates of relapse to cocaine use in humans after prolonged abstinence indicated that the drug-induced adaptation in cellular mechanisms was persistent during abstinence (Lu et al., 2004). A time-dependent increase in cocaine seeking induced by cocaine cues involves activation of ERK signaling, suggesting this pathway mediates the incubation of cocaine craving (Lu et al., 2005). Together, we suggest that long-lasting changes of the ERK signaling pathway in the PFC induced by cocaine treatment may be one of the most important mechanisms underlying cocaine addiction.

### Fluctuant activity of plasticity-related pathways after cocaine withdrawal

In the present study, we found that long-term potentiation (LTP), the regulation of the actin cytoskeleton, and the axon guidance pathway demonstrated fluctuant changes after withdrawal of repeated cocaine treatment, consistent with the current opinion that during abstinence, neuroplasticity at many levels, ranging from cellular alterations to morphological and structural changes, plays an essential role in addictive behaviors (Kasanetz et al., 2012; Nyberg, 2014). Repeated cocaine exposure modifies the induction of tetanus-induced LTP *in vitro* and *in vivo*, which was associated with dysfunction of glutamate receptors (Yao et al., 2004; Goto and Grace, 2005). The actin cytoskeleton has been shown to promote presynaptic vesicle movement, postsynaptic glutamate receptor trafficking, and morphogenesis of dendritic spines (Nyberg, 2014). In addition, changes in dendritic spine diameter and density due to chronic cocaine administration were associated with a deteriorating actin cytoskeleton and reduction in glutamate signaling-related proteins (Shen et al., 2009). However, alterations of drug-induced synaptic plasticity were guided by molecular cues, such as axon guidance molecules, that form projection-target connections during rearrangement of the synapse (Bahi and Dreyer, 2005). Together with these results, our findings indicate that potential associations among these varied levels of neuroplasticity play an important role in cortical reorganization after withdrawal of repeated cocaine treatment.

### Common and different changes of gene expression in cocaine responds across different brain regions

Previous studies suggest that circadian genes, including *Per1, Per2*, and *Npas2* in NAc and hippocampus play roles in modulating cocaine reward or sensitization (Abarca et al., 2002; Imbesi et al., 2009). In dorsal striatum, >20% (29 genes total) were identified as genes associated with the circadian system in a total of 139 genes, which were regulated by cocaine self-administration (Lynch et al., 2008). Together with our findings of circadian pathway enrichment in the PFC after repeated cocaine treatment, it is indicated that circadian genes play essential roles in cocaine addiction regardless of treatment paradigm or brain regions.

As mentioned above, MAPK pathway also plays important roles in cocaine addiction across different brain regions. There is evidence indicating that MAPKs have roles in the circadian biological clock. MAPK signaling pathways and circadian clocks affect similar biological processes and defects in either pathway lead to many of the same types of human diseases (Goldsmith and Bell-Pedersen, 2013). Interestingly, our further analysis showed that MAPK and circadian pathway had similar dynamic changes after cocaine withdrawal (*r* > 0.70, *p* < 0.05), indicating that interaction between these two pathways mediated cocaine-induced neuroadaptations.

On another hand, a study compared changes of genes expression with cocaine abstinence between the mPFC and NAc (Freeman et al., 2010). The results showed that only a limited number of changes were observed in both brain regions. Another study showed that genes involved in cholinergic, glutamatergic, and GABAergic signaling transmissions in NAc were in response to chronic cocaine exposure and withdrawal (Eipper-Mains et al., 2013). In our study, only a few neurotransmitter receptors such as Grin2a and Grin2b, were changed in the PFC 7d after cocaine withdrawal. These inconsistent results may reflect distinct roles of genes in different brain regions. In addition, different cocaine treatment paradigms may contribute to these differences. For example, repeated cocaine treatment resulted in different changes of gene network with or without locomotor sensitization test (Eipper-Mains et al., 2013; Feng et al., 2014).

Repeated exposure to cocaine leads to sensitization, and may persist for weeks to years after cessation of drug taking, which may contribute to drug relapse (Hyman et al., 2006). Our finding indicates that maladaptive neural plasticity after repeated cocaine treatment is an ongoing, degenerative process with dynamic changes of the gene network at different stages of withdrawal. The long-lasting adverse effects on neuropsychiatric impairments that persist following abstinence lead to poor treatment outcomes. Understanding the dynamic profile of gene expression after abstinence could be helpful in developing new therapeutic approaches according to different periods of abstinence.

## Author contributions

Conceived and designed the experiment: MZ; Performed the experiment: ML, PX, and YX; Analyzed the data: WT and HT; Contributed reagents/materials/analysis tools: MZ; Wrote and Revised the manuscript: QD and MZ.

## Funding

This work was supported by the National Natural Science Foundation of China (No. 30870821; No. 91132728); and Key Laboratory of Mental Health, Institute of Psychology, Chinese Academy of Sciences.

### Conflict of interest statement

The authors declare that the research was conducted in the absence of any commercial or financial relationships that could be construed as a potential conflict of interest.
