# Supplementary material for: Dynamic Expression Changes in the Transcriptome of the Prefrontal Cortex after Repeated Exposure to Cocaine in Mice
Source: Front Pharmacol. 2017 Mar 23;8:142. doi: 10.3389/fphar.2017.00142 (PMC5362609; doi:10.3389/fphar.2017.00142)
Supplement: Supplementary file 1 [file Table1.DOCX]

Supplementary

Table S1. primers used for qRT-PCR analyses.

| Gene | Sequence of primer (5’-3’) | |
| --- | --- | --- |
|  | Forward | Reverse |
| *JunB* | AGCAGGGCGACTTGAGGA | GGTGATACTGGCAATTATGC |
| *Nr4a1* | TCGTGTCAGCACTATGGG | GTCCGTACAACTTCCTTCACC |
| *Nr4a2* | ACTATTCCAGGTTCCAGGCA | AAGATGAGTTTACCCTCCACTG |
| *Nr4a3*  *Kdm6b*  *Notch1*  *Notch2*  *Wnt4*  *Wnt7b*  *Grin2b* | GGTTAAGGAAGTTGTGCGT  GAAATTCCGAGAGTCCTACCT  ACAGTGCAACCCCCTGTATG  AGCAGGAGGTGATAGGCTCT  GCAGGAAGGCCATCTTGACAC  GGCAATCTGAGCAATTGTGG  ACCTCCTGTGTGAGAGGAAATC | CATTCATCATACAGATCGGAGG  CCTAAGTTGAGCCGAAGTG  CCGCAGAAAGTGGAAGGAGT  TGGGCGTTTCTTGGACTCTC  GGCTTGAACTGTGCATTCCG  TCCAGAACCTTTCTGCCC  GGATGCCGGGGATAGAAAGG |
