# Supplementary material for: Dynamic Expression Changes in the Transcriptome of the Prefrontal Cortex after Repeated Exposure to Cocaine in Mice
Source: Front Pharmacol. 2017 Mar 23;8:142. doi: 10.3389/fphar.2017.00142 (PMC5362609; doi:10.3389/fphar.2017.00142)
Supplement: Supplementary file 2 [file Table2.DOCX]

Supplementary

Table S2. The DEGs involved in 2 hours of withdrawal after chronic cocaine treatment.

| gene | Saline  FPKM | Cocaine  FPKM | log2  (fold_change) | significant |
| --- | --- | --- | --- | --- |
| 1190002N15Rik | 13.2688 | 8.4088 | -0.65807 | yes |
| 2810459M11Rik | 2.66031 | 4.46339 | 0.746546 | yes |
| 6820431F20Rik,AC152164.1,  Gm15319 | 14.2939 | 8.17925 | -0.80536 | yes |
| A830073O21Rik | 1.369 | 0.759966 | -0.84912 | yes |
| A930011G23Rik,Rasgef1b | 30.6366 | 15.3003 | -1.00169 | yes |
| Ackr3 | 3.19135 | 4.98039 | 0.642093 | Yes |
| Adcy9 | 10.0344 | 13.1781 | 0.393191 | Yes |
| Adra2c | 5.52141 | 7.38698 | 0.419949 | Yes |
| Adrbk1 | 71.948 | 82.8637 | 0.203787 | Yes |
| Agpat4 | 16.703 | 20.8199 | 0.317857 | Yes |
| Ahdc1 | 7.81718 | 12.0845 | 0.628436 | Yes |
| AI593442 | 43.6691 | 31.7899 | -0.45805 | Yes |
| Ak4 | 12.0803 | 15.9901 | 0.404513 | Yes |
| Aldoc,Pigs | 516.493 | 603.859 | 0.225462 | Yes |
| Alox12b | 2.31913 | 4.25189 | 0.874521 | Yes |
| Amer2 | 4.1001 | 5.20303 | 0.343694 | Yes |
| Ankrd33b | 10.7595 | 14.4333 | 0.423792 | Yes |
| Ankrd34c | 2.45425 | 1.44421 | -0.76501 | Yes |
| Ankrd6 | 7.60419 | 10.5196 | 0.468208 | Yes |
| Ankrd63 | 7.34266 | 10.6375 | 0.53478 | Yes |
| Apod | 103.696 | 125.043 | 0.270059 | Yes |
| Arhgap31 | 3.21006 | 4.07744 | 0.345065 | Yes |
| Arl5b | 2.64539 | 3.73231 | 0.496587 | Yes |
| B630005N14Rik | 6.36364 | 8.13378 | 0.354075 | Yes |
| Baz2a | 3.64149 | 4.87679 | 0.421403 | Yes |
| Bcl6 | 3.58043 | 5.95184 | 0.733203 | Yes |
| Bcl9l | 4.28033 | 5.79186 | 0.436303 | Yes |
| Bmp3 | 3.95995 | 2.94538 | -0.42703 | Yes |
| Bok | 41.0716 | 49.1617 | 0.259395 | Yes |
| Brinp2 | 27.2786 | 31.8818 | 0.224963 | Yes |
| Bsn | 34.4586 | 41.1193 | 0.254954 | Yes |
| C130074G19Rik | 35.9418 | 29.5219 | -0.28388 | Yes |
| C77080 | 5.56383 | 8.3398 | 0.583934 | Yes |
| Cacnb1 | 40.109 | 49.26 | 0.296488 | Yes |
| Calhm2 | 1.47403 | 3.42412 | 1.21597 | Yes |
| Calr | 139.512 | 162.926 | 0.223829 | Yes |
| Camk1g | 13.5751 | 20.7592 | 0.612783 | Yes |
| Caskin1 | 14.9036 | 18.378 | 0.302317 | Yes |
| Cbl | 4.27776 | 5.20802 | 0.283879 | Yes |
| Ccdc6 | 6.10766 | 7.67544 | 0.32963 | Yes |
| Ccnd1 | 14.6704 | 17.709 | 0.271582 | Yes |
| Ccnd2 | 10.3529 | 18.3123 | 0.822775 | Yes |
| Cdc42ep3 | 7.31674 | 5.14984 | -0.50667 | Yes |
| Cdk5r1 | 78.4136 | 94.9424 | 0.275947 | Yes |
| Cds1 | 26.4055 | 21.0931 | -0.32406 | Yes |
| Cecr6 | 14.9773 | 18.7728 | 0.325869 | Yes |
| Chrm4 | 6.95633 | 12.319 | 0.824492 | Yes |
| Chst1 | 87.7159 | 109.169 | 0.315654 | Yes |
| Cirbp | 54.2426 | 43.2396 | -0.32707 | Yes |
| Clic4 | 12.2002 | 15.9724 | 0.388674 | Yes |
| Coq10b | 14.6554 | 20.3431 | 0.473112 | Yes |
| Cotl1 | 27.9388 | 34.7208 | 0.313528 | Yes |
| Cox6a1 | 670.314 | 754.721 | 0.171108 | Yes |
| Cplx2 | 202.234 | 231.056 | 0.192218 | Yes |
| Creld2 | 6.552 | 9.18853 | 0.487898 | Yes |
| Crkl | 6.3757 | 8.46326 | 0.408629 | Yes |
| Cry1 | 4.05395 | 6.04352 | 0.576063 | Yes |
| Cry2 | 26.83 | 32.6676 | 0.284015 | Yes |
| Csrnp1 | 1.62235 | 3.89423 | 1.26325 | Yes |
| Cst3 | 1525.49 | 1727.82 | 0.179686 | Yes |
| Ctdsp2 | 13.9847 | 17.6077 | 0.332359 | Yes |
| Ctgf | 2.32025 | 3.7982 | 0.71104 | yes |
| D8Ertd82e | 8.78341 | 11.0124 | 0.326275 | yes |
| Dact2 | 46.9645 | 35.2502 | -0.41394 | yes |
| Dagla | 16.8322 | 20.6226 | 0.293006 | yes |
| Ddit4 | 10.3171 | 13.3723 | 0.374206 | yes |
| Ddit4l | 15.8045 | 10.0955 | -0.64663 | yes |
| Dlc1 | 6.71504 | 8.95941 | 0.416009 | yes |
| Dnajb1 | 23.4201 | 29.1791 | 0.317188 | yes |
| Dnajb4 | 20.3258 | 27.1315 | 0.416661 | yes |
| Dnajb5 | 45.9743 | 78.7746 | 0.776903 | yes |
| Dpf1 | 15.9105 | 25.4213 | 0.676057 | yes |
| Dpy19l1 | 31.3615 | 25.4888 | -0.29913 | yes |
| Dscaml1 | 4.41965 | 5.78951 | 0.389512 | yes |
| Dusp18 | 7.31925 | 9.00203 | 0.298556 | yes |
| Dusp4 | 1.37768 | 5.02303 | 1.86632 | yes |
| Dusp5 | 1.7701 | 4.49577 | 1.34474 | yes |
| DXBay18 | 0.239391 | 0.650892 | 1.44305 | yes |
| Efnb1 | 4.61585 | 6.6786 | 0.53295 | yes |
| Egln2 | 29.1998 | 35.4916 | 0.281519 | yes |
| Egr3 | 35.6727 | 45.5904 | 0.353912 | yes |
| Egr4 | 10.7826 | 22.8967 | 1.08643 | yes |
| Eif4ebp2 | 8.08865 | 10.6232 | 0.393241 | yes |
| Eif5 | 59.3805 | 49.9765 | -0.24874 | yes |
| Elavl3 | 29.1489 | 34.3319 | 0.236108 | yes |
| Elfn2 | 3.79176 | 6.7429 | 0.830501 | yes |
| Elfn2 | 14.1511 | 22.2909 | 0.655543 | yes |
| Elmsan1 | 1.91082 | 2.95483 | 0.62888 | yes |
| Eltd1 | 5.24671 | 8.05485 | 0.618445 | yes |
| Emx1 | 19.7871 | 13.7045 | -0.52991 | yes |
| Enc1 | 153.184 | 204.338 | 0.415688 | yes |
| Ep300 | 4.91939 | 6.20745 | 0.335522 | yes |
| Ephb3 | 6.24759 | 9.95976 | 0.672811 | yes |
| Etnppl | 4.00779 | 6.80897 | 0.764629 | yes |
| Ets2 | 15.1864 | 18.0739 | 0.251122 | yes |
| Etv5 | 20.1705 | 26.4101 | 0.388837 | yes |
| Ezr | 22.8201 | 26.733 | 0.22832 | yes |
| Fam101b | 6.36402 | 4.61508 | -0.46358 | yes |
| Fam107a | 126.442 | 162.096 | 0.358369 | yes |
| Fam180a | 2.50059 | 4.38409 | 0.81001 | yes |
| Fam212b | 32.3252 | 37.5459 | 0.215995 | yes |
| Fam43b | 6.88532 | 10.9763 | 0.672796 | yes |
| Fam53c | 11.1923 | 13.9703 | 0.319853 | yes |
| Fam65a | 15.3629 | 20.0917 | 0.387148 | yes |
| Fbxo32 | 1.3872 | 0.911009 | -0.60664 | yes |
| Fbxo34 | 23.5226 | 17.6563 | -0.41386 | yes |
| Fbxw7 | 58.8964 | 48.158 | -0.2904 | yes |
| Fem1c | 5.91378 | 4.39204 | -0.42919 | yes |
| Fkbp5 | 5.93107 | 9.35245 | 0.657053 | yes |
| Flrt2 | 13.2774 | 8.16594 | -0.70128 | yes |
| Fmnl1 | 26.3875 | 43.9576 | 0.736258 | yes |
| Fnbp1l | 13.5813 | 10.8925 | -0.31829 | yes |
| Fosb | 1.55734 | 3.64383 | 1.22637 | yes |
| Foxo6 | 2.87234 | 4.82284 | 0.747656 | yes |
| Frmd6 | 5.6819 | 7.99333 | 0.492422 | yes |
| Furin | 5.00893 | 7.36647 | 0.556472 | yes |
| Fus | 101.948 | 80.0236 | -0.34934 | yes |
| Fzd1 | 2.74101 | 3.71243 | 0.437657 | yes |
| Fzd2 | 3.60161 | 4.97381 | 0.465709 | yes |
| Gadd45b | 5.28924 | 9.03361 | 0.772242 | yes |
| Gal3st3 | 19.6271 | 25.4405 | 0.374276 | yes |
| Git1 | 113.635 | 128.759 | 0.180261 | yes |
| Gjb6 | 27.9956 | 37.7735 | 0.432175 | yes |
| Glul | 638.538 | 736.073 | 0.205076 | yes |
| Gm12840 | 1.70848 | 10.7782 | 2.65732 | yes |
| Gm1604b,Rps6ka2 | 15.9347 | 20.9218 | 0.392839 | yes |
| Gm16229 | 3.97958 | 8.50292 | 1.09534 | yes |
| Gm26809 | 4.28155 | 9.67072 | 1.17549 | yes |
| Gm26917 | 463.431 | 604.094 | 0.382418 | yes |
| Gm27032,Mir124a-3 | 4.15068 | 2.17446 | -0.93269 | yes |
| Gm27300,Meg3,Mir1906-1,  Mir770 | 28.0993 | 16.5227 | -0.76609 | yes |
| Gm4631 | 20.2182 | 16.9361 | -0.25555 | yes |
| Golph3 | 29.0395 | 34.2162 | 0.236664 | yes |
| Gpr17 | 6.83422 | 9.63887 | 0.496087 | yes |
| Gpr37l1 | 86.5234 | 98.5721 | 0.188089 | yes |
| Gpr68 | 2.87964 | 5.25656 | 0.868228 | yes |
| Gprin1 | 30.0727 | 38.4035 | 0.35278 | yes |
| Gpt2 | 11.8129 | 17.9142 | 0.600732 | yes |
| Hbb-bs | 112.53 | 169.235 | 0.588725 | yes |
| Hbb-bt | 40.4215 | 66.7587 | 0.723831 | yes |
| Hecw2 | 7.848 | 5.24564 | -0.58121 | yes |
| Herpud1 | 25.2415 | 32.6553 | 0.37152 | yes |
| Hes5 | 10.5386 | 14.7588 | 0.485882 | yes |
| Hexim1 | 11.5721 | 15.6142 | 0.432204 | yes |
| Hif1an | 4.33239 | 5.49122 | 0.341963 | yes |
| Hlf | 41.1771 | 49.8479 | 0.275689 | yes |
| Hnrnpdl | 57.1655 | 45.7382 | -0.32174 | yes |
| Homer1 | 35.8964 | 45.1985 | 0.332434 | yes |
| Hs3st1 | 12.4098 | 7.67034 | -0.69412 | yes |
| Hspa5 | 76.2876 | 108.508 | 0.508286 | yes |
| Hspb1 | 5.04943 | 9.7289 | 0.946157 | yes |
| Hspb8 | 8.30221 | 10.9241 | 0.395951 | yes |
| Htr6 | 3.01622 | 1.9673 | -0.61652 | yes |
| Hunk | 4.04131 | 2.14425 | -0.91435 | yes |
| Id1 | 17.3338 | 24.8928 | 0.522143 | yes |
| Id3 | 22.8855 | 30.8233 | 0.429589 | yes |
| Id4 | 23.4005 | 31.2038 | 0.41518 | yes |
| Ier2 | 2.83086 | 6.87177 | 1.27944 | yes |
| Ier3 | 7.12579 | 11.0204 | 0.62905 | yes |
| Ier5 | 20.9962 | 36.4073 | 0.794102 | yes |
| Ier5l | 5.82 | 11.628 | 0.998512 | yes |
| Igsf5,Pcp4 | 246.447 | 211.211 | -0.22259 | yes |
| Inf2 | 22.9338 | 18.5454 | -0.30642 | yes |
| Inhbb | 1.75311 | 3.32431 | 0.923137 | yes |
| Irf2bpl | 16.4644 | 28.9752 | 0.815469 | yes |
| Irs2 | 4.74183 | 8.49717 | 0.841536 | yes |
| Irs2 | 3.68891 | 9.45272 | 1.35753 | yes |
| Islr2 | 13.1989 | 23.178 | 0.812339 | yes |
| Itgb5 | 14.9943 | 18.035 | 0.266389 | yes |
| Itm2c | 383.534 | 432.007 | 0.171702 | yes |
| Itpkb | 5.23202 | 7.26549 | 0.473692 | yes |
| Jade2 | 6.99597 | 11.8551 | 0.760917 | yes |
| Junb | 11.3395 | 61.3821 | 2.43647 | yes |
| Kcnf1 | 25.74 | 32.8064 | 0.349962 | yes |
| Kcng1 | 5.87648 | 9.02237 | 0.618555 | yes |
| Kcnh3 | 54.4458 | 64.4193 | 0.242672 | yes |
| Kcnj12 | 6.03936 | 8.49512 | 0.492238 | yes |
| Kcnj2 | 5.50036 | 3.38295 | -0.70125 | yes |
| Kctd10 | 16.1961 | 19.6251 | 0.277053 | yes |
| Kdm2a | 7.20459 | 9.55368 | 0.40714 | yes |
| Kdm5b | 6.74394 | 8.5573 | 0.343565 | yes |
| Kdm6b | 1.19951 | 2.44117 | 1.02512 | yes |
| Kdm7a | 3.60705 | 4.98483 | 0.466724 | yes |
| Klf2 | 5.13836 | 8.54586 | 0.733918 | yes |
| Klf4 | 2.05639 | 4.21097 | 1.03404 | yes |
| Klf9 | 33.292 | 39.8963 | 0.261081 | yes |
| Ldhd | 9.89115 | 12.7921 | 0.371043 | yes |
| Lingo1 | 155.649 | 183.114 | 0.234442 | yes |
| Lman2 | 15.1245 | 18.6002 | 0.298433 | yes |
| Lrrn2 | 23.6967 | 30.2342 | 0.351495 | yes |
| Lrrtm2 | 8.13428 | 10.849 | 0.415471 | yes |
| Lrtm2 | 18.4296 | 22.0361 | 0.257847 | yes |
| Luzp1 | 8.20765 | 11.023 | 0.425481 | yes |
| Lyst | 2.78301 | 3.42773 | 0.300606 | yes |
| Lzts3 | 49.2615 | 61.2528 | 0.314317 | yes |
| Mapk11 | 14.3802 | 11.1678 | -0.36474 | yes |
| Mef2d | 29.4947 | 37.9138 | 0.36227 | yes |
| Mertk | 8.25063 | 11.398 | 0.466203 | yes |
| Mettl7a1 | 8.65919 | 13.4394 | 0.63416 | yes |
| Mfge8 | 93.9771 | 108.178 | 0.203021 | yes |
| Mfn1 | 11.2297 | 14.7115 | 0.38963 | yes |
| Mfsd2a | 9.47511 | 14.8807 | 0.651226 | yes |
| Mgp | 71.4159 | 94.8195 | 0.408937 | yes |
| Midn | 10.5973 | 19.4016 | 0.872472 | yes |
| Mkl2 | 20.6246 | 24.8787 | 0.270543 | yes |
| Mknk2 | 8.96125 | 12.2096 | 0.446242 | yes |
| Mlc1 | 68.8498 | 77.9498 | 0.179093 | yes |
| Mllt1 | 19.4438 | 22.9885 | 0.241603 | yes |
| Mmd2 | 38.175 | 44.7017 | 0.227701 | yes |
| Mnt | 6.36272 | 8.37662 | 0.396724 | yes |
| Mt1 | 319.04 | 394.569 | 0.306541 | yes |
| Mt2 | 216.513 | 263.424 | 0.282932 | yes |
| mt-Rnr1 | 6129.51 | 7314.53 | 0.254993 | yes |
| mt-Rnr2 | 4699.41 | 5710.32 | 0.281093 | yes |
| Mxd4 | 22.9401 | 28.3276 | 0.304334 | yes |
| Myadm | 16.0168 | 20.231 | 0.336979 | yes |
| Mycn | 1.91247 | 4.2587 | 1.15498 | yes |
| Mylip | 3.91061 | 5.35758 | 0.454186 | yes |
| Myrip | 12.3597 | 15.0883 | 0.287782 | yes |
| N4bp1 | 5.95535 | 7.54902 | 0.342102 | yes |
| Nab2 | 5.69318 | 12.3401 | 1.11605 | yes |
| Nedd9 | 1.19196 | 2.29189 | 0.943202 | yes |
| Neurod2 | 20.4067 | 25.4612 | 0.31926 | yes |
| Nostrin | 1.97383 | 3.71763 | 0.913388 | yes |
| Notch2 | 1.66646 | 2.4415 | 0.550981 | yes |
| Nptx1 | 51.6807 | 68.4276 | 0.404954 | yes |
| Nr4a2 | 1.43053 | 3.01638 | 1.07627 | yes |
| Nr4a3 | 1.81652 | 5.48296 | 1.59378 | yes |
| Nuak1 | 13.6642 | 21.3942 | 0.646823 | yes |
| Nudt18 | 13.2485 | 10.909 | -0.28031 | yes |
| Omg | 68.2709 | 54.6997 | -0.31974 | yes |
| Otub2 | 11.4076 | 17.072 | 0.581633 | yes |
| Otud1 | 9.21249 | 15.4627 | 0.747128 | yes |
| P4ha1 | 9.56204 | 14.5579 | 0.606413 | yes |
| Pak4 | 12.5917 | 15.8035 | 0.32777 | yes |
| Pcdh1 | 26.5576 | 39.3636 | 0.567738 | yes |
| Pcdh20 | 4.6054 | 3.29048 | -0.48503 | yes |
| Pcdh8 | 7.66822 | 10.2331 | 0.416283 | yes |
| Pdgfb | 7.80571 | 13.3421 | 0.773381 | yes |
| Pdia4 | 9.91752 | 15.0982 | 0.606327 | yes |
| Pdia6 | 39.163 | 50.1125 | 0.355678 | yes |
| Pdp1 | 43.6308 | 64.6611 | 0.567553 | yes |
| Per1 | 9.0585 | 21.5437 | 1.24992 | yes |
| Pfkfb3 | 4.81394 | 6.9482 | 0.529422 | yes |
| Pitpnm2 | 38.9945 | 53.9098 | 0.467277 | yes |
| Plat | 8.08882 | 11.0352 | 0.448117 | yes |
| Plk2 | 53.1642 | 63.5623 | 0.257717 | yes |
| Plk3 | 2.69852 | 5.3626 | 0.990763 | yes |
| Plxna1 | 9.70336 | 11.9641 | 0.302158 | yes |
| Pnrc1 | 16.5773 | 23.3906 | 0.49672 | yes |
| Pou3f1 | 7.16175 | 10.2525 | 0.517587 | yes |
| Ppargc1b | 0.945907 | 1.32008 | 0.480859 | yes |
| Ppp1r3c | 15.9321 | 20.9454 | 0.394696 | yes |
| Prdm2 | 6.45942 | 8.47977 | 0.39262 | yes |
| Prdx6 | 91.2964 | 110.685 | 0.277832 | yes |
| Prelp | 4.45935 | 5.86053 | 0.394197 | yes |
| Prickle2 | 25.2731 | 29.451 | 0.220714 | yes |
| Ptgds | 1820.24 | 2089.17 | 0.198803 | yes |
| Ptpn1 | 8.01398 | 11.9686 | 0.578666 | yes |
| Pvrl3 | 13.511 | 9.84015 | -0.45738 | yes |
| Pyurf | 17.2648 | 6.00669 | -1.52319 | yes |
| Rab3d | 2.61582 | 3.98442 | 0.60711 | yes |
| Rapgef1 | 19.4101 | 23.5691 | 0.280087 | yes |
| Rara | 1.70067 | 3.82789 | 1.17044 | yes |
| Rasl10b | 100.233 | 119.803 | 0.257308 | yes |
| Rasl11a | 1.22829 | 3.49199 | 1.50739 | yes |
| Rasl11b | 44.876 | 29.9974 | -0.58111 | yes |
| Rbm24 | 5.57337 | 8.18179 | 0.553867 | yes |
| Rcan1 | 25.7194 | 32.8774 | 0.35424 | yes |
| Rhbdl3 | 2.33629 | 3.88696 | 0.734422 | yes |
| Rhobtb2 | 18.8185 | 23.9515 | 0.347962 | yes |
| Rilpl1 | 31.796 | 43.9396 | 0.466676 | yes |
| Rilpl2 | 5.90794 | 9.28884 | 0.652842 | yes |
| Rnf112 | 49.644 | 39.9148 | -0.3147 | yes |
| Rnf165 | 8.36342 | 10.8673 | 0.37783 | yes |
| Rsrp1 | 21.2284 | 37.8138 | 0.832916 | yes |
| Rtn4rl2 | 32.6387 | 42.6012 | 0.38431 | yes |
| Rundc1 | 9.67794 | 13.1377 | 0.440942 | yes |
| Rybp | 10.055 | 12.506 | 0.314707 | yes |
| S1pr1 | 63.6241 | 75.0699 | 0.23866 | yes |
| Sall2 | 7.53584 | 9.57853 | 0.346036 | yes |
| Sbk1 | 13.5971 | 23.9298 | 0.815512 | yes |
| Schip1 | 112.625 | 131.056 | 0.218661 | yes |
| Sde2 | 4.95376 | 6.42934 | 0.376147 | yes |
| Sdf2l1 | 5.36767 | 10.8027 | 1.00903 | yes |
| Sdpr | 4.00504 | 5.59319 | 0.481854 | yes |
| Selplg | 11.7648 | 14.2378 | 0.275246 | yes |
| Serpinh1 | 8.19779 | 11.3102 | 0.464314 | yes |
| Sertad1 | 5.54609 | 10.3499 | 0.900069 | yes |
| Sertm1 | 15.6461 | 12.55 | -0.31811 | yes |
| Sesn3 | 14.8839 | 17.6622 | 0.246917 | yes |
| Setd1b | 2.43387 | 3.54343 | 0.54189 | yes |
| Setd8 | 11.1254 | 15.5056 | 0.478933 | yes |
| Sgk1 | 18.6211 | 14.2107 | -0.38996 | yes |
| Sgsm1 | 14.1311 | 17.6496 | 0.320765 | yes |
| Siah3 | 1.09406 | 1.85071 | 0.75839 | yes |
| Sik1 | 1.04681 | 3.16811 | 1.59762 | yes |
| Ski | 30.9988 | 37.3729 | 0.26978 | yes |
| Slc24a4 | 6.32631 | 7.92442 | 0.324941 | yes |
| Slc25a25 | 13.01 | 16.5279 | 0.345286 | yes |
| Slc25a44 | 22.3602 | 26.841 | 0.263504 | yes |
| Slc2a1 | 35.9753 | 48.8285 | 0.440715 | yes |
| Slc30a3 | 76.9735 | 89.5183 | 0.217821 | yes |
| Slc36a1 | 8.74136 | 11.3323 | 0.374506 | yes |
| Slc7a14 | 9.91593 | 12.7019 | 0.357225 | yes |
| Slitrk1 | 26.7559 | 22.4475 | -0.2533 | yes |
| Smad3 | 14.0807 | 19.3879 | 0.461442 | yes |
| Sntb2 | 1.32562 | 1.91121 | 0.527819 | yes |
| Socs3 | 0.609053 | 1.43547 | 1.23688 | yes |
| Soga1 | 2.18272 | 2.66265 | 0.286733 | yes |
| Sowaha | 24.9714 | 33.2721 | 0.414037 | yes |
| Sox18 | 5.20622 | 7.5876 | 0.543405 | yes |
| Sox2 | 14.8053 | 18.1665 | 0.295167 | yes |
| Sox8 | 9.0628 | 14.3457 | 0.662587 | yes |
| Spon1 | 5.49478 | 4.40558 | -0.31873 | yes |
| Spred1 | 32.2957 | 42.3118 | 0.389721 | yes |
| Spred2 | 19.2202 | 28.1882 | 0.552468 | yes |
| Sprn | 68.0123 | 57.8958 | -0.23234 | yes |
| Spry3 | 1.48033 | 2.47629 | 0.742258 | yes |
| Spry4 | 1.35776 | 3.02372 | 1.1551 | yes |
| Spty2d1 | 2.48152 | 3.67118 | 0.56502 | yes |
| Srsf5 | 67.5726 | 47.8564 | -0.49773 | yes |
| St6gal2 | 11.4168 | 7.53985 | -0.59856 | yes |
| Stk40 | 6.22279 | 11.4441 | 0.878968 | yes |
| Stmn4 | 175.96 | 150.978 | -0.22091 | yes |
| Sult1a1 | 6.2814 | 9.68876 | 0.625226 | yes |
| Tcerg1 | 11.0348 | 8.7058 | -0.34202 | yes |
| Tgfb3 | 3.1419 | 4.45867 | 0.504975 | yes |
| Timp3 | 21.3613 | 26.7172 | 0.322765 | yes |
| Tiparp | 2.20542 | 4.28826 | 0.959339 | yes |
| Tipin | 9.7068 | 6.11736 | -0.66609 | yes |
| Tjp1 | 11.6288 | 14.6323 | 0.331459 | yes |
| Tle3 | 8.79994 | 12.5026 | 0.506667 | yes |
| Tmem28 | 3.34158 | 4.44614 | 0.412021 | yes |
| Tmtc3 | 1.52984 | 2.198 | 0.522804 | yes |
| Tnfrsf11b | 0.938768 | 1.66462 | 0.826349 | yes |
| Tnfrsf21 | 13.6145 | 17.4224 | 0.3558 | yes |
| Tnik | 10.6691 | 13.5742 | 0.347426 | yes |
| Tns3 | 8.82219 | 11.6791 | 0.404714 | yes |
| Trak1 | 37.034 | 48.585 | 0.39166 | yes |
| Trank1 | 7.77144 | 4.99343 | -0.63815 | yes |
| Trib1 | 3.07221 | 8.76947 | 1.51321 | yes |
| Trib2 | 9.00401 | 13.5976 | 0.594709 | yes |
| Tsc22d1 | 215.088 | 252.752 | 0.232793 | yes |
| Tsc22d3 | 21.9307 | 30.7992 | 0.489942 | yes |
| Tspan14 | 14.0609 | 17.6759 | 0.330092 | yes |
| Ttyh3 | 41.0194 | 49.6753 | 0.276221 | yes |
| Ulk1 | 13.0025 | 15.8435 | 0.285102 | yes |
| Unc119b | 6.24311 | 8.18821 | 0.391283 | yes |
| Unc5a | 18.0657 | 22.3612 | 0.307738 | yes |
| Usp2 | 25.4246 | 15.9098 | -0.67631 | yes |
| Vcl | 2.09192 | 3.13529 | 0.583772 | yes |
| Vgf | 81.0128 | 104.644 | 0.369263 | yes |
| Vps37b | 13.8219 | 16.6791 | 0.27108 | yes |
| Vwa1 | 8.61069 | 12.1781 | 0.500088 | yes |
| Wdtc1 | 13.4991 | 17.0076 | 0.333315 | yes |
| Wipf2 | 10.3724 | 13.3993 | 0.3694 | yes |
| Wipf3 | 28.3654 | 37.7131 | 0.410932 | yes |
| Wisp1 | 0.50886 | 1.45076 | 1.51147 | yes |
| Wnt10a | 5.86277 | 9.59957 | 0.711387 | yes |
| Wnt4 | 6.46228 | 8.63219 | 0.417682 | yes |
| Wnt9a | 7.83304 | 5.34092 | -0.55248 | yes |
| Xbp1 | 32.6077 | 43.0829 | 0.401903 | yes |
| Xdh | 0.946541 | 1.60252 | 0.759603 | yes |
| Zbtb7a | 15.5354 | 18.6003 | 0.259767 | yes |
| Zfp180 | 10.7218 | 13.4387 | 0.325847 | yes |
| Zfp35 | 5.51698 | 7.05844 | 0.355469 | yes |
| Zfp366 | 1.06203 | 1.78473 | 0.748873 | yes |
| Zfp36l2 | 5.92323 | 8.11906 | 0.454929 | yes |
| Zfp598 | 4.37822 | 5.84998 | 0.418086 | yes |
| Zfp651 | 10.8773 | 13.5088 | 0.312571 | yes |
| Zfp703 | 11.6443 | 14.5233 | 0.318742 | yes |
| Zfp831 | 2.02419 | 2.85117 | 0.494209 | yes |
| Zmiz1 | 12.6951 | 15.814 | 0.316936 | yes |
| Zswim4 | 2.6361 | 4.17569 | 0.66361 | yes |
| Arl4d | 5.94736 | 12.5629 | 1.07885 | yes |
| Bhlhe40 | 36.0611 | 80.5689 | 1.15978 | yes |
| Egr2 | 0.465105 | 5.69993 | 3.61532 | yes |
| Klf10 | 6.27838 | 16.9553 | 1.43327 | yes |
| Per2 | 2.57253 | 5.981 | 1.2172 | yes |
| Adcy1 | 52.7886 | 59.5437 | 0.173724 | yes |
| Ago2 | 4.00081 | 5.27327 | 0.398406 | yes |
| Alkbh5 | 7.69673 | 9.63003 | 0.323294 | yes |
| Ankrd52 | 5.21713 | 6.54022 | 0.326084 | yes |
| Atp1a2 | 209.048 | 247.108 | 0.241311 | yes |
| Bcr | 15.1072 | 18.0947 | 0.260334 | yes |
| Btg2 | 2.80657 | 5.86984 | 1.06451 | yes |
| Cdc42bpb | 23.1233 | 26.5797 | 0.200978 | yes |
| Chst2 | 19.6482 | 22.3505 | 0.185909 | yes |
| Cmtm4 | 16.4563 | 19.376 | 0.235626 | yes |
| Crtc1 | 30.4501 | 36.3771 | 0.256583 | yes |
| Dio2 | 22.9053 | 30.1839 | 0.3981 | yes |
| Dtl | 2.22538 | 1.47898 | -0.58946 | yes |
| Dusp1 | 5.73448 | 24.0606 | 2.06894 | yes |
| Fbxl18 | 3.68041 | 4.6876 | 0.348982 | yes |
| Fosl2 | 2.79636 | 4.98237 | 0.833282 | yes |
| Gatsl2 | 13.0646 | 16.4131 | 0.329191 | yes |
| Gfod1 | 17.263 | 24.933 | 0.530376 | yes |
| Gm15800 | 10.602 | 12.5142 | 0.23923 | yes |
| Gm26917,Yam1 | 359.475 | 477.674 | 0.410137 | yes |
| Gstm1 | 121.79 | 140.556 | 0.206759 | yes |
| Hba-a1 | 75.3838 | 117.702 | 0.642807 | yes |
| Hba-a2 | 93.2225 | 147.22 | 0.659222 | yes |
| Homer2 | 7.18488 | 8.67922 | 0.272601 | yes |
| Igf1r | 3.36623 | 4.10812 | 0.287345 | yes |
| Igsf9b | 2.32765 | 3.24011 | 0.477167 | yes |
| Irf2bp2 | 5.6501 | 7.69636 | 0.445899 | yes |
| Klf13 | 21.7639 | 26.7666 | 0.298495 | yes |
| Lars2 | 583.471 | 716.615 | 0.296537 | yes |
| Lix1l | 6.8577 | 8.8821 | 0.373175 | yes |
| Lrrc58 | 8.88569 | 11.1041 | 0.321541 | yes |
| Lzts1 | 19.9216 | 24.032 | 0.270627 | yes |
| Map1a | 120.47 | 136.968 | 0.185168 | yes |
| Mast3 | 47.382 | 55.0097 | 0.215347 | yes |
| Mir6236 | 18994 | 24168.8 | 0.347599 | yes |
| Mir6236 | 26.3649 | 8.0377 | -1.71377 | yes |
| Mn1 | 5.20992 | 7.85408 | 0.592181 | yes |
| Mt3 | 414.94 | 472.831 | 0.188422 | yes |
| Myh9 | 8.75242 | 10.7495 | 0.29652 | yes |
| Ncan | 27.9561 | 31.6762 | 0.180234 | yes |
| Ncs1 | 60.6575 | 68.6002 | 0.177526 | yes |
| Npas4 | 1.14271 | 2.54481 | 1.15509 | yes |
| Palm | 69.6651 | 81.4529 | 0.22553 | yes |
| Paqr8 | 22.8795 | 30.3227 | 0.40634 | yes |
| Pdxk | 22.7882 | 26.0145 | 0.191027 | yes |
| Plxnd1 | 14.9675 | 17.9697 | 0.26373 | yes |
| Pnmal2 | 65.8028 | 74.0234 | 0.169831 | yes |
| Pom121 | 9.43257 | 11.3965 | 0.272872 | yes |
| Ppp1r16b | 13.9992 | 16.7326 | 0.25732 | yes |
| Prr12 | 6.00902 | 7.53067 | 0.325649 | yes |
| Ptrf | 8.06551 | 10.4662 | 0.375895 | yes |
| Pvrl1 | 5.54598 | 7.64391 | 0.462868 | yes |
| Rims4 | 18.234 | 22.1983 | 0.28382 | yes |
| Rps14 | 382.09 | 455.503 | 0.253548 | yes |
| Rps21 | 646.07 | 738.633 | 0.193166 | yes |
| Rps29 | 665.164 | 774.595 | 0.219734 | yes |
| Sccpdh | 45.2747 | 36.7961 | -0.29915 | yes |
| Scrt1 | 30.1717 | 36.8514 | 0.288524 | yes |
| Sdc4 | 16.2405 | 20.9684 | 0.368617 | yes |
| Sema3g | 1.2537 | 1.96068 | 0.645163 | yes |
| Sft2d2 | 7.33114 | 9.0468 | 0.303371 | yes |
| Shank2 | 19.6594 | 23.2049 | 0.239215 | yes |
| Shank3 | 37.1765 | 45.2231 | 0.282671 | yes |
| Sipa1l1 | 53.905 | 64.4142 | 0.25696 | yes |
| Snhg11 | 34.3643 | 20.6569 | -0.73429 | yes |
| Socs7 | 15.2318 | 18.4207 | 0.274247 | yes |
| Spry2 | 14.0251 | 19.2502 | 0.45687 | yes |
| Stox2 | 9.29732 | 11.3985 | 0.293951 | yes |
| Synpo | 53.8306 | 76.7195 | 0.511165 | yes |
| Thbd | 6.41403 | 9.00361 | 0.489274 | yes |
| Tmcc2 | 43.9869 | 54.1008 | 0.298578 | yes |
| Tmem151b | 22.5295 | 32.4527 | 0.526528 | yes |
| Traf3 | 6.03854 | 8.10513 | 0.424635 | yes |
| Ttr | 1.89483 | 0.46031 | -2.04138 | yes |
| Wfs1 | 45.3509 | 56.1692 | 0.308649 | yes |
| Zranb2 | 37.5681 | 30.9757 | -0.27837 | yes |
| Arc | 5.57495 | 77.387 | 3.79506 | yes |
| Dusp6 | 14.1202 | 32.5138 | 1.2033 | yes |
| Egr1 | 41.632 | 120.711 | 1.5358 | yes |
| Fos | 2.01558 | 14.231 | 2.81977 | yes |
| Nr4a1 | 8.011 | 36.4151 | 2.18448 | yes |
