# Supplementary material for: Dynamic Expression Changes in the Transcriptome of the Prefrontal Cortex after Repeated Exposure to Cocaine in Mice
Source: Front Pharmacol. 2017 Mar 23;8:142. doi: 10.3389/fphar.2017.00142 (PMC5362609; doi:10.3389/fphar.2017.00142)
Supplement: Supplementary file 3 [file Table3.DOCX]

Supplementary

Table S3 The DEGs involved in 24 hours of withdrawal after chronic cocaine treatment.

| gene | Saline  FPKM | Cocaine  FPKM | log2  (fold_change) | significant |
| --- | --- | --- | --- | --- |
| Foxp4 | 30.7852 | 10.383 | -1.56801 | yes |
| Hist1h1c | 15.5597 | 10.2986 | -0.59537 | Yes |
| Zfp185 | 0.399021 | 12.6891 | 4.99098 | Yes |
| Arl4d | 6.24447 | 10.493 | 0.74878 | Yes |
| Bhlhe40 | 28.0388 | 48.1011 | 0.778645 | Yes |
| Egr2 | 0.711922 | 2.29304 | 1.68747 | Yes |
| Klf10 | 8.83267 | 13.0117 | 0.558889 | Yes |
| Per2 | 1.27367 | 3.08517 | 1.27636 | Yes |
| Gm22405 | 105.119 | 46.3667 | -1.18086 | Yes |
| Arc | 23.0593 | 53.9336 | 1.22583 | Yes |
| Dusp6 | 18.281 | 25.6758 | 0.490065 | Yes |
| Egr1 | 34.8967 | 69.8103 | 1.00035 | Yes |
| Fos | 3.93219 | 9.21691 | 1.22895 | yes |
| Nr4a1 | 15.2016 | 24.8133 | 0.706887 | yes |
