# Supplementary material for: Dynamic Expression Changes in the Transcriptome of the Prefrontal Cortex after Repeated Exposure to Cocaine in Mice
Source: Front Pharmacol. 2017 Mar 23;8:142. doi: 10.3389/fphar.2017.00142 (PMC5362609; doi:10.3389/fphar.2017.00142)
Supplement: Supplementary file 4 [file Table4.DOCX]

Supplementary

Table S4. The DEGs involved in 7days of withdrawal after chronic cocaine treatment.

| gene | Saline  FPKM | | | Cocaine  FPKM | | log2  (fold_change) | significant |
| --- | --- | --- | --- | --- | --- | --- | --- |
| 0610009B22Rik | | 23.6669 | | | 15.7308 | -0.58928 | Yes |
| 1110058L19Rik | | 36.4401 | | | 26.4297 | -0.46337 | Yes |
| 1500011B03Rik,2610524H06Rik,  Gm20499 | | | 94.1308 | | 77.17 | -0.28663 | Yes |
| 1500011K16Rik | | | 51.7498 | | 38.3267 | -0.4332 | Yes |
| 1700001P01Rik,Rpl23 | | | 258.269 | | 199.184 | -0.37477 | Yes |
| 1810022K09Rik | | | 62.0676 | | 38.572 | -0.68628 | Yes |
| 1810037I17Rik | | | 53.595 | | 42.2725 | -0.34238 | Yes |
| 2010107E04Rik | | | 374.952 | | 270.817 | -0.46939 | Yes |
| 2700060E02Rik | | | 45.8369 | | 37.557 | -0.28743 | Yes |
| 6330403K07Rik | | | 200.696 | | 167.034 | -0.26488 | Yes |
| Abhd2 | | | 15.5116 | | 18.2943 | 0.238049 | Yes |
| AC127338.1,Gm5776 | | | 50.4154 | | 36.5212 | -0.46513 | Yes |
| AC148327.1,Gm5559 | | | 348.062 | | 271.994 | -0.35577 | Yes |
| Acot11 | | | 6.5104 | | 8.89745 | 0.450646 | Yes |
| Acta2 | | | 6.71167 | | 9.3992 | 0.485866 | Yes |
| Adcy5 | | | 17.8007 | | 21.4755 | 0.270759 | Yes |
| Add2 | | | 20.4027 | | 24.4243 | 0.259553 | Yes |
| Agap1 | | | 5.63062 | | 7.08216 | 0.330895 | Yes |
| Agap2 | | | 167.297 | | 212.304 | 0.343716 | Yes |
| Ak6,Taf9 | | | 77.883 | | 61.999 | -0.32906 | Yes |
| Akr1e1 | | | 18.6108 | | 14.2817 | -0.38197 | Yes |
| Ap2s1 | | | 185.519 | | 155.249 | -0.25698 | Yes |
| Ap3s1 | | | 45.447 | | 35.4177 | -0.35971 | Yes |
| Apba1 | | | 18.6489 | | 23.1774 | 0.313629 | Yes |
| Apc2 | | | 10.1056 | | 12.2263 | 0.274835 | Yes |
| Arf3 | | | 340.056 | | 427.108 | 0.328829 | Yes |
| Arhgap32 | | | 31.8902 | | 38.087 | 0.256185 | Yes |
| Arhgap33 | | | 31.367 | | 36.8201 | 0.231245 | Yes |
| Arhgap35 | | | 15.9012 | | 20.2026 | 0.34541 | Yes |
| Arhgef12 | | | 12.1615 | | 15.97 | 0.393045 | Yes |
| Arpc3 | | | 183.949 | | 154.634 | -0.25045 | Yes |
| Arpc5l | | | 71.1024 | | 57.9417 | -0.2953 | Yes |
| Atp1b2 | | | 129.965 | | 154.533 | 0.249793 | Yes |
| Atp5e | | | 321.223 | | 254.951 | -0.33336 | yes |
| Atp5f1 | | | 189.725 | | 154.274 | -0.29841 | yes |
| Atp5j | | | 252.598 | | 193.938 | -0.38125 | yes |
| Atp5j2 | | | 213.978 | | 151.853 | -0.49479 | yes |
| Atp5l | | | 280.743 | | 214.648 | -0.38728 | yes |
| Atp6v0b,Dph2 | | | 247.743 | | 198.651 | -0.31861 | yes |
| Atp6v1c1 | | | 143.57 | | 122.299 | -0.23135 | yes |
| Atp6v1d | | | 200.86 | | 169.22 | -0.24729 | yes |
| Atp6v1f | | | 180.078 | | 149.346 | -0.26996 | yes |
| Atp6v1g1 | | | 81.9441 | | 67.0116 | -0.29023 | yes |
| Atp6v1h | | | 97.5176 | | 79.6875 | -0.29131 | yes |
| Atpif1 | | | 397.835 | | 288.044 | -0.46588 | yes |
| Axl | | | 11.0502 | | 14.175 | 0.35928 | yes |
| B4galt5 | | | 7.67291 | | 9.47874 | 0.304922 | yes |
| Bcan | | | 51.7642 | | 63.5422 | 0.295761 | yes |
| Bloc1s2 | | | 27.5016 | | 20.7658 | -0.40531 | yes |
| Bnip3 | | | 79.3313 | | 64.8052 | -0.29178 | yes |
| Bre,Mrpl33 | | | 110.623 | | 84.926 | -0.38137 | yes |
| Btbd17 | | | 14.0561 | | 19.0302 | 0.437095 | yes |
| Btbd3 | | | 38.1084 | | 46.301 | 0.280934 | yes |
| Bud31 | | | 40.358 | | 29.6186 | -0.44635 | yes |
| Caln1 | | | 0.781951 | | 2.32377 | 1.57132 | yes |
| Camk1 | | | 56.323 | | 46.806 | -0.26703 | yes |
| Canx | | | 68.1371 | | 86.3111 | 0.341105 | yes |
| Cbr1 | | | 44.7836 | | 37.3279 | -0.26271 | yes |
| Ccdc142,Mrpl53 | | | 51.1072 | | 37.3458 | -0.45258 | yes |
| Ccdc59 | | | 11.5305 | | 8.49813 | -0.44023 | yes |
| Cd248 | | | 1.7239 | | 2.71849 | 0.657131 | yes |
| Cd83 | | | 10.6769 | | 8.46965 | -0.33412 | yes |
| Cdk5r2 | | | 146.257 | | 177.417 | 0.27864 | yes |
| Cdr1 | | | 5.26874 | | 14.3323 | 1.44374 | yes |
| Cds2 | | | 46.0772 | | 56.6075 | 0.296941 | yes |
| Cenpv | | | 21.0123 | | 14.8716 | -0.49868 | yes |
| Cep170b | | | 33.2074 | | 39.7562 | 0.259679 | yes |
| Chchd10 | | | 99.0136 | | 119.288 | 0.268748 | yes |
| Chchd2 | | | 299.228 | | 233.968 | -0.35494 | yes |
| Cib2 | | | 21.5689 | | 17.3412 | -0.31475 | yes |
| Clic5 | | | 0.940785 | | 1.41979 | 0.593738 | yes |
| Cmss1 | | | 4051.45 | | 1259.28 | -1.68584 | yes |
| Cntnap1 | | | 31.705 | | 36.9415 | 0.220531 | yes |
| Coa6 | | | 24.8761 | | 17.7146 | -0.48982 | yes |
| Col1a1 | | | 1.75132 | | 2.54144 | 0.5372 | yes |
| Col1a2 | | | 4.70246 | | 6.1352 | 0.383694 | yes |
| Commd3 | | | 68.1856 | | 50.1179 | -0.44414 | yes |
| Commd4 | | | 37.4366 | | 29.7917 | -0.32954 | yes |
| Cops5 | | | 54.6981 | | 42.8281 | -0.35293 | yes |
| Cox6c | | | 695.164 | | 528.396 | -0.39573 | yes |
| Cox7a2 | | | 306.73 | | 217.238 | -0.49769 | yes |
| Cox7b | | | 173.783 | | 129.835 | -0.4206 | yes |
| Cox7c | | | 344.903 | | 252.27 | -0.45123 | yes |
| Cpsf2 | | | 74.0787 | | 55.8638 | -0.40714 | yes |
| Crym | | | 46.4765 | | 37.8991 | -0.29434 | yes |
| Cst6 | | | 1.51101 | | 0.94368 | -0.67914 | yes |
| Cstb | | | 39.7512 | | 31.1236 | -0.35299 | yes |
| Ctnnd2 | | | 32.6515 | | 39.268 | 0.266206 | yes |
| Cycs | | | 72.5307 | | 54.6069 | -0.40951 | yes |
| Cyr61 | | | 1.23738 | | 2.24548 | 0.859733 | yes |
| D17Wsu92e | | | 64.3418 | | 78.4884 | 0.286722 | yes |
| Dact3 | | | 68.1663 | | 82.5266 | 0.275801 | yes |
| Deb1 | | | 59.5704 | | 44.7373 | -0.41312 | yes |
| Dgkh | | | 1.41343 | | 2.68062 | 0.923366 | yes |
| Dld | | | 53.9686 | | 43.2959 | -0.31789 | yes |
| Dnajc15 | | | 46.7211 | | 33.808 | -0.46671 | yes |
| Dpysl2 | | | 30.7225 | | 36.8292 | 0.261552 | yes |
| Dynll1 | | | 396.158 | | 301.773 | -0.39261 | yes |
| Dynlrb1 | | | 273.852 | | 211.673 | -0.37157 | yes |
| Dynlt3 | | | 75.6142 | | 62.9782 | -0.2638 | yes |
| Eef1e1 | | | 23.4755 | | 18.2723 | -0.36149 | yes |
| Eif2a | | | 20.4748 | | 15.5152 | -0.40017 | yes |
| Eif3f | | | 37.0211 | | 45.2288 | 0.288896 | yes |
| Emc2 | | | 56.9051 | | 44.0555 | -0.36924 | yes |
| Enah | | | 11.4027 | | 14.1928 | 0.315786 | yes |
| Epas1 | | | 20.3694 | | 25.8099 | 0.34152 | yes |
| Erdr1 | | | 35.7907 | | 18.325 | -0.96577 | yes |
| Fads2 | | | 40.3569 | | 48.3429 | 0.260488 | yes |
| Fam103a1 | | | 60.4089 | | 50.2086 | -0.26683 | yes |
| Fam162a | | | 38.4138 | | 29.4239 | -0.38464 | yes |
| Fat4 | | | 0.549919 | | 0.77997 | 0.5042 | yes |
| Fau | | | 390.951 | | 301.066 | -0.37691 | yes |
| Fbxo41 | | | 17.2347 | | 20.4572 | 0.247299 | yes |
| Fgfr3 | | | 17.9165 | | 24.0944 | 0.42741 | yes |
| Filip1l | | | 22129.5 | | 12854.6 | -0.78369 | yes |
| Fkbp3 | | | 153.261 | | 114.222 | -0.42415 | yes |
| Flt1 | | | 9.07394 | | 11.4524 | 0.335854 | yes |
| Fmod | | | 10.2979 | | 12.8499 | 0.319402 | yes |
| Fn3krp | | | 9.5284 | | 12.4774 | 0.389008 | yes |
| Fxyd1,Fxyd7 | | | 137.458 | | 109.974 | -0.32183 | yes |
| Fzd4 | | | 1.24682 | | 1.71582 | 0.460644 | yes |
| Gja1 | | | 61.3146 | | 74.1922 | 0.275037 | yes |
| Gjb2 | | | 6.16633 | | 7.9648 | 0.369227 | yes |
| Glrx3 | | | 48.1773 | | 39.8597 | -0.27343 | yes |
| Gm10020 | | | 17.7727 | | 11.9848 | -0.56845 | yes |
| Gm10040 | | | 6.87955 | | 9.13719 | 0.409438 | yes |
| Gm10053 | | | 41.1877 | | 31.8489 | -0.37097 | yes |
| Gm10123 | | | 78.5246 | | 58.8048 | -0.41721 | yes |
| Gm10221 | | | 46.6698 | | 32.5701 | -0.51894 | yes |
| Gm11407 | | | 6.62651 | | 2.31931 | -1.51456 | yes |
| Gm11808 | | | 98.1297 | | 68.7424 | -0.51349 | yes |
| Gm12895 | | | 87.6544 | | 185.319 | 1.08011 | yes |
| Gm12896 | | | 87.8733 | | 184.932 | 1.0735 | yes |
| Gm14236 | | | 18.0194 | | 7.79036 | -1.20979 | yes |
| Gm14539 | | | 92.861 | | 24.6069 | -1.91601 | yes |
| Gm15662 | | | 398.059 | | 153.349 | -1.37617 | yes |
| Gm17275 | | | 41.8513 | | 30.1015 | -0.47544 | yes |
| Gm17494,Mir760 | | | 9.49025 | | 6.31965 | -0.5866 | yes |
| Gm20538,Ndufb8,Sec31b | | | 246.098 | | 207.303 | -0.2475 | yes |
| Gm21887 | | | 26.1296 | | 14.7599 | -0.824 | yes |
| Gm21897 | | | 2.54982 | | 4.06738 | 0.673705 | yes |
| Gm21967,Gm7120 | | | 23.4161 | | 15.0839 | -0.63449 | yes |
| Gm22179 | | | 1.71039 | | 14.8352 | 3.11662 | yes |
| Gm26191 | | | 41.0706 | | 162.071 | 1.98044 | yes |
| Gm26549 | | | 4.39519 | | 0.899316 | -2.28902 | yes |
| Gm26778 | | | 9.17904 | | 11.5591 | 0.332613 | yes |
| Gm4540 | | | 26.4124 | | 15.4986 | -0.76908 | yes |
| Gm9843 | | | 27.4817 | | 16.1527 | -0.7667 | yes |
| Gng13 | | | 112.51 | | 80.9178 | -0.47552 | yes |
| Gng5 | | | 59.1963 | | 42.4791 | -0.47876 | yes |
| Gpr56 | | | 12.3442 | | 16.56 | 0.423873 | yes |
| Gpx4 | | | 448.691 | | 360.713 | -0.31487 | yes |
| Grin2a | | | 5.48557 | | 7.41956 | 0.435691 | yes |
| Grin2b | | | 10.2133 | | 14.2297 | 0.478458 | yes |
| Gspt1 | | | 3.35595 | | 6.53878 | 0.9623 | yes |
| Gtf3c1 | | | 9.21829 | | 11.1476 | 0.274164 | yes |
| Gtl3 | | | 27.5716 | | 21.4543 | -0.36191 | yes |
| Hcfc1r1 | | | 114.886 | | 92.7992 | -0.30802 | yes |
| Hddc2 | | | 16.8266 | | 12.2877 | -0.45352 | yes |
| Hepacam | | | 17.8412 | | 23.2197 | 0.380135 | yes |
| Higd1a | | | 42.0433 | | 34.2012 | -0.29783 | yes |
| Higd2a | | | 134.883 | | 109.942 | -0.29497 | yes |
| Hint1 | | | 277.365 | | 225.042 | -0.30159 | yes |
| Hipk2 | | | 4.71478 | | 6.86616 | 0.542313 | yes |
| Hmgn3 | | | 43.2804 | | 32.0456 | -0.43359 | yes |
| Hopx | | | 31.9468 | | 24.6468 | -0.37427 | yes |
| Hprt | | | 118.513 | | 99.165 | -0.25715 | yes |
| Hs6st3 | | | 1.62371 | | 3.10498 | 0.935288 | yes |
| Hsbp1 | | | 184.7 | | 157.32 | -0.23149 | yes |
| Ighm | | | 6958.89 | | 11.5333 | -9.23691 | yes |
| Irs1 | | | 0.273988 | | 0.53279 | 0.959454 | yes |
| Iscu | | | 67.1612 | | 55.5732 | -0.27324 | yes |
| Itpa | | | 45.3093 | | 35.1794 | -0.36508 | yes |
| Itpr1 | | | 54.2319 | | 64.7901 | 0.256632 | yes |
| Kbtbd11 | | | 13.7363 | | 17.1918 | 0.323721 | yes |
| Kcna2 | | | 4.09096 | | 6.22168 | 0.604865 | yes |
| Kcnip4 | | | 35.2567 | | 28.0157 | -0.33166 | yes |
| Khsrp | | | 11.6507 | | 14.0876 | 0.27401 | yes |
| Kif1c | | | 17.7508 | | 22.3156 | 0.330172 | yes |
| Klf12 | | | 0.328583 | | 1.01427 | 1.62612 | yes |
| Klhl24 | | | 6.2657 | | 7.63152 | 0.284493 | yes |
| Krt12 | | | 9.64016 | | 6.91732 | -0.47884 | yes |
| Ksr2 | | | 2.24359 | | 3.3647 | 0.584671 | yes |
| Kxd1,Uba52 | | | 707.509 | | 538.351 | -0.3942 | yes |
| Lamb2 | | | 4.17083 | | 5.54913 | 0.411926 | yes |
| Laptm5 | | | 15.5418 | | 20.1882 | 0.377356 | yes |
| Ldlr | | | 2.9232 | | 3.78307 | 0.372006 | yes |
| Lifr | | | 3.19493 | | 4.10673 | 0.362206 | yes |
| Lmtk2 | | | 16.0748 | | 19.6393 | 0.288944 | yes |
| Lnpep | | | 3.68934 | | 5.69701 | 0.626841 | yes |
| Lrp4 | | | 3.84987 | | 5.87138 | 0.60889 | yes |
| Lrrc17 | | | 4.97076 | | 1.00984 | -2.29934 | yes |
| Lsm3 | | | 27.0091 | | 19.4877 | -0.47088 | yes |
| Ly6h | | | 181.676 | | 148.983 | -0.28622 | yes |
| Lyrm4 | | | 20.8385 | | 15.7272 | -0.40599 | yes |
| Malat1 | | | 12.3744 | | 36.9057 | 1.57649 | yes |
| Man2a2 | | | 15.7808 | | 18.9122 | 0.261146 | yes |
| Map3k9 | | | 3.02772 | | 3.91765 | 0.371754 | yes |
| Mapk4 | | | 13.7368 | | 16.732 | 0.284561 | yes |
| Marf1 | | | 13.3838 | | 18.1361 | 0.438383 | yes |
| Mblac2 | | | 8.58181 | | 6.81376 | -0.33283 | yes |
| Mcts1 | | | 61.1284 | | 45.0675 | -0.43976 | yes |
| Mdh1 | | | 461.52 | | 381.34 | -0.27532 | yes |
| Med13l | | | 5.77397 | | 7.77828 | 0.429887 | yes |
| Med30 | | | 24.1374 | | 17.4888 | -0.46484 | yes |
| Megf8 | | | 16.5714 | | 20.4198 | 0.301271 | yes |
| Mien1 | | | 89.0867 | | 70.8857 | -0.32972 | yes |
| Minos1 | | | 135.706 | | 103.147 | -0.39579 | yes |
| Mllt6 | | | 15.242 | | 20.1276 | 0.401121 | yes |
| Mpc1 | | | 169.869 | | 136.161 | -0.31911 | yes |
| Mpc2 | | | 94.7258 | | 69.3797 | -0.44924 | yes |
| Mrpl11 | | | 15.2075 | | 11.8765 | -0.35668 | yes |
| Mrpl20 | | | 84.0554 | | 64.1037 | -0.39093 | yes |
| Mrpl30 | | | 53.8699 | | 42.4559 | -0.34352 | yes |
| Mrpl36 | | | 32.9728 | | 24.7953 | -0.41121 | yes |
| Mrpl41 | | | 57.1597 | | 44.9287 | -0.34736 | yes |
| Mrpl42 | | | 79.4361 | | 57.9593 | -0.45475 | yes |
| Mrpl50 | | | 16.5114 | | 13.4102 | -0.30014 | yes |
| Mrpl54 | | | 39.4002 | | 27.6039 | -0.51333 | yes |
| Mrps18c | | | 39.9626 | | 27.939 | -0.51637 | yes |
| Mrps9 | | | 17.3757 | | 13.8318 | -0.32908 | yes |
| Msi2 | | | 11.5215 | | 14.1278 | 0.294212 | yes |
| mt-Nd3 | | | 8645.83 | | 6450.22 | -0.42266 | yes |
| Mtss1l | | | 30.1444 | | 38.124 | 0.33881 | yes |
| Myeov2 | | | 186.496 | | 151.576 | -0.29911 | yes |
| Myl4 | | | 68.0202 | | 48.0649 | -0.50098 | yes |
| Myl6 | | | 453.838 | | 382.63 | -0.24623 | yes |
| N6amt2 | | | 30.7749 | | 23.4121 | -0.3945 | yes |
| Naca | | | 139.098 | | 110.477 | -0.33235 | yes |
| Nacc2 | | | 12.3684 | | 14.9595 | 0.274395 | yes |
| Nae1 | | | 21.6765 | | 17.0531 | -0.3461 | yes |
| Nat8l | | | 51.6853 | | 63.1921 | 0.289991 | yes |
| Ndufa1 | | | 208.631 | | 164.651 | -0.34154 | yes |
| Ndufa11 | | | 19.8779 | | 16.5565 | -0.26377 | yes |
| Ndufa13,Yjefn3 | | | 122.232 | | 95.9523 | -0.34923 | yes |
| Ndufa2 | | | 194.837 | | 152.751 | -0.35109 | yes |
| Ndufa3 | | | 291.451 | | 230.118 | -0.34088 | yes |
| Ndufa4 | | | 668.064 | | 490.12 | -0.44685 | yes |
| Ndufa5 | | | 200.82 | | 146.816 | -0.45189 | yes |
| Ndufa6 | | | 196.626 | | 147.116 | -0.4185 | yes |
| Ndufb11 | | | 156.955 | | 123.323 | -0.3479 | yes |
| Ndufb4 | | | 71.778 | | 57.6873 | -0.31529 | yes |
| Ndufb5 | | | 172.036 | | 120.326 | -0.51576 | yes |
| Ndufb6 | | | 110.799 | | 87.1749 | -0.34596 | yes |
| Ndufb7 | | | 123.682 | | 100.315 | -0.30209 | yes |
| Ndufc2 | | | 193.966 | | 139.721 | -0.47326 | yes |
| Ndufs3 | | | 115.76 | | 89.6711 | -0.36842 | yes |
| Ndufs4 | | | 56.8702 | | 42.7257 | -0.41257 | yes |
| Ndufs5 | | | 216.192 | | 177.506 | -0.28445 | yes |
| Ndufv2 | | | 124.467 | | 95.7268 | -0.37877 | yes |
| Nedd8 | | | 179.034 | | 150.512 | -0.25036 | yes |
| Neurl1a | | | 57.1687 | | 72.559 | 0.34393 | yes |
| Neurl1b | | | 6.59828 | | 8.88351 | 0.429039 | yes |
| Neurod6 | | | 37.2634 | | 28.0841 | -0.40801 | yes |
| Nlgn2 | | | 37.2385 | | 43.7935 | 0.233923 | yes |
| Nmnat2 | | | 24.1485 | | 28.7189 | 0.250067 | yes |
| Nop10 | | | 43.757 | | 32.4119 | -0.43299 | yes |
| Notch3 | | | 1.05644 | | 1.54825 | 0.55142 | yes |
| Nova2 | | | 6.7051 | | 9.45102 | 0.495213 | yes |
| Nprl2 | | | 17.8001 | | 14.0652 | -0.33976 | yes |
| Nrn1 | | | 238.247 | | 204.314 | -0.22167 | yes |
| Ntsr2 | | | 44.7141 | | 55.3208 | 0.307092 | yes |
| Oaz1 | | | 326.784 | | 266.49 | -0.29426 | yes |
| Ostc | | | 39.4128 | | 29.7474 | -0.4059 | yes |
| Paip2 | | | 70.8245 | | 59.7426 | -0.24549 | yes |
| Pbxip1 | | | 10.0912 | | 12.9301 | 0.357631 | yes |
| Pcsk1n | | | 202.241 | | 238.776 | 0.239583 | yes |
| Pdcd10 | | | 31.6427 | | 24.2975 | -0.38106 | yes |
| Pdcd5 | | | 56.9554 | | 41.5957 | -0.4534 | yes |
| Pet100 | | | 13.4375 | | 9.08073 | -0.56539 | yes |
| Pfdn1 | | | 47.3951 | | 39.4648 | -0.26417 | yes |
| Pgk1-rs7 | | | 21.7266 | | 17.8856 | -0.28066 | yes |
| Plxna4 | | | 3.78042 | | 4.69501 | 0.31258 | yes |
| Pnck | | | 29.9873 | | 23.3611 | -0.36024 | yes |
| Podxl | | | 5.44578 | | 7.25155 | 0.41315 | yes |
| Polr1d | | | 55.1529 | | 43.6858 | -0.33627 | yes |
| Polr2a | | | 7.64236 | | 9.27046 | 0.278624 | yes |
| Polr2f | | | 75.0646 | | 53.5987 | -0.48594 | yes |
| Polr3k | | | 15.3421 | | 12.6811 | -0.27482 | yes |
| Pomp | | | 168.243 | | 142.8 | -0.23655 | yes |
| Ppa1 | | | 59.1042 | | 49.1274 | -0.26673 | yes |
| Ppdpf | | | 68.9415 | | 47.908 | -0.52511 | yes |
| Ppia | | | 1570.57 | | 1119.65 | -0.48824 | yes |
| Ppid | | | 35.6455 | | 28.9964 | -0.29785 | yes |
| Ppp1r12b | | | 8.38997 | | 11.0783 | 0.401004 | yes |
| Ppp1r9b | | | 209.492 | | 273.673 | 0.385562 | yes |
| Prdx1 | | | 152.909 | | 114.886 | -0.41247 | yes |
| Prdx2 | | | 151.616 | | 126.132 | -0.2655 | yes |
| Prdx4 | | | 18.4215 | | 13.243 | -0.47616 | yes |
| Prkcb | | | 10.6106 | | 20.3888 | 0.942267 | yes |
| Psenen | | | 91.285 | | 63.7102 | -0.51885 | yes |
| Psma1 | | | 67.1963 | | 54.0839 | -0.31318 | yes |
| Psma2 | | | 98.6046 | | 81.1144 | -0.2817 | yes |
| Psma4 | | | 81.494 | | 63.2481 | -0.36567 | yes |
| Psma6 | | | 82.6382 | | 66.7178 | -0.30874 | yes |
| Psma7 | | | 103.188 | | 83.1274 | -0.31188 | yes |
| Psmb1 | | | 136.576 | | 109.761 | -0.31534 | yes |
| Psmb5 | | | 127.16 | | 100.957 | -0.3329 | yes |
| Psmb6 | | | 118.016 | | 95.2578 | -0.30907 | yes |
| Psmb7 | | | 123.588 | | 102.703 | -0.26705 | yes |
| Ptcd2 | | | 17.9095 | | 13.909 | -0.36471 | yes |
| Ptprb | | | 3.16428 | | 4.26907 | 0.432046 | yes |
| Ptprj | | | 13.9032 | | 17.074 | 0.296383 | yes |
| Ptprz1 | | | 26.7086 | | 32.2828 | 0.273459 | yes |
| Pttg1 | | | 64.0242 | | 45.9024 | -0.48005 | yes |
| Pygb | | | 39.9371 | | 48.2191 | 0.271876 | yes |
| Rab11fip4 | | | 29.2375 | | 38.8417 | 0.409785 | yes |
| Rabac1 | | | 164.688 | | 141.202 | -0.22197 | yes |
| Rere | | | 9.09615 | | 11.9669 | 0.395721 | yes |
| Resp18 | | | 111.473 | | 82.8786 | -0.42762 | yes |
| Rfx3 | | | 3.6138 | | 4.65604 | 0.365588 | yes |
| Rit2 | | | 54.3698 | | 44.6639 | -0.2837 | yes |
| Romo1 | | | 95.325 | | 67.5632 | -0.49662 | yes |
| RP23-410L16.1 | | | 9.10292 | | 5.332 | -0.77165 | yes |
| RP23-8J15.5 | | | 8946.49 | | 6701.14 | -0.41692 | yes |
| Rpl10a | | | 172.976 | | 141.326 | -0.29154 | yes |
| Rpl11 | | | 237.99 | | 193.956 | -0.29518 | yes |
| Rpl15 | | | 173.977 | | 134.135 | -0.37521 | yes |
| Rpl15-ps2 | | | 27.0948 | | 18.3963 | -0.5586 | yes |
| Rpl15-ps3 | | | 41.8067 | | 29.2263 | -0.51646 | yes |
| Rpl17 | | | 364.682 | | 277.235 | -0.39553 | yes |
| Rpl18a | | | 162.84 | | 136.005 | -0.25979 | yes |
| Rpl22l1 | | | 168.701 | | 137.044 | -0.29983 | yes |
| Rpl23a | | | 462.179 | | 383.952 | -0.26752 | yes |
| Rpl26 | | | 684.495 | | 555.673 | -0.30081 | yes |
| Rpl27 | | | 162.578 | | 134.428 | -0.2743 | yes |
| Rpl31 | | | 250.139 | | 186.688 | -0.42211 | yes |
| Rpl31-ps14 | | | 89.3108 | | 66.5856 | -0.42363 | yes |
| Rpl32 | | | 339.795 | | 281.947 | -0.26924 | yes |
| Rpl35 | | | 151.294 | | 111.087 | -0.44567 | yes |
| Rpl35a | | | 339.417 | | 275.667 | -0.30014 | yes |
| Rpl36 | | | 359.187 | | 288.797 | -0.31468 | yes |
| Rpl36al | | | 151.632 | | 123.321 | -0.29815 | yes |
| Rpl37 | | | 246.208 | | 193.34 | -0.34873 | yes |
| Rpl37a | | | 277.768 | | 220.069 | -0.33592 | yes |
| Rpl38 | | | 470.278 | | 378.252 | -0.31417 | yes |
| Rpl39 | | | 281.033 | | 208.865 | -0.42817 | yes |
| Rpl41 | | | 907.027 | | 680.373 | -0.41482 | yes |
| Rpl7 | | | 235.349 | | 193.91 | -0.27941 | yes |
| Rpl9 | | | 380.756 | | 303.517 | -0.32709 | yes |
| Rps15 | | | 1037.72 | | 857.753 | -0.27479 | yes |
| Rps17 | | | 367.731 | | 299.143 | -0.29782 | yes |
| Rps19bp1 | | | 21.1472 | | 16.1542 | -0.38856 | yes |
| Rps20 | | | 371.483 | | 277.781 | -0.41935 | yes |
| Rps23 | | | 288.64 | | 225.668 | -0.35507 | yes |
| Rps24 | | | 713.742 | | 583.747 | -0.29006 | yes |
| Rps27 | | | 649.085 | | 409.393 | -0.66492 | yes |
| Rps27a | | | 269.557 | | 206.1 | -0.38725 | yes |
| Rps3a1 | | | 308.48 | | 252.386 | -0.28954 | yes |
| Rps5 | | | 287.901 | | 244.457 | -0.23599 | yes |
| Rps7 | | | 260.19 | | 196.832 | -0.4026 | yes |
| Rsl1d1 | | | 24.0969 | | 19.8605 | -0.27895 | yes |
| Rwdd1 | | | 33.4586 | | 26.9864 | -0.31015 | yes |
| S100a10 | | | 52.176 | | 40.6725 | -0.35933 | yes |
| Scand1 | | | 26.2955 | | 34.3289 | 0.384604 | yes |
| Scd1 | | | 39.8815 | | 48.0532 | 0.268914 | yes |
| Scfd1 | | | 14.1998 | | 11.4359 | -0.3123 | yes |
| Scg5 | | | 179.549 | | 149.688 | -0.26242 | yes |
| Scn2b | | | 42.9657 | | 51.0047 | 0.247444 | yes |
| Sdhaf1 | | | 4.37266 | | 2.69705 | -0.69713 | yes |
| Sdhb | | | 115.702 | | 97.7278 | -0.24357 | yes |
| Sec61b | | | 45.5609 | | 34.846 | -0.38681 | yes |
| Sec61g | | | 44.9552 | | 30.8828 | -0.54169 | yes |
| Selk | | | 75.5902 | | 59.7742 | -0.33868 | yes |
| Sema6b | | | 26.1737 | | 31.0117 | 0.244692 | yes |
| Sf3b6 | | | 43.3737 | | 29.7758 | -0.54268 | yes |
| Sh3glb1 | | | 14.5362 | | 19.0259 | 0.388315 | yes |
| Sh3pxd2a | | | 4.02011 | | 5.3648 | 0.416291 | yes |
| Shank1 | | | 39.5746 | | 67.5387 | 0.771139 | yes |
| Shc3 | | | 4.27213 | | 6.9588 | 0.703883 | yes |
| Shc3 | | | 13.582 | | 17.3237 | 0.351057 | yes |
| Sla2 | | | 8.81559 | | 6.13108 | -0.52392 | yes |
| Slc25a4 | | | 394.941 | | 333.745 | -0.24289 | yes |
| Slc4a4 | | | 14.2937 | | 18.018 | 0.334057 | yes |
| Slc6a1 | | | 73.0284 | | 87.272 | 0.257063 | yes |
| Slc6a11 | | | 28.845 | | 36.1097 | 0.324067 | yes |
| Slc7a2 | | | 3.43142 | | 4.71412 | 0.458183 | yes |
| Slitrk2 | | | 3.65015 | | 4.8979 | 0.424205 | yes |
| Snrpb2 | | | 20.6495 | | 13.6517 | -0.59703 | yes |
| Snrpd1 | | | 56.6666 | | 43.7255 | -0.37403 | yes |
| Snrpd2 | | | 131.733 | | 95.4903 | -0.46419 | yes |
| Snx2 | | | 29.2469 | | 23.9667 | -0.28725 | yes |
| Snx22 | | | 8.79208 | | 6.41126 | -0.4556 | yes |
| Sox9 | | | 5.06328 | | 6.49435 | 0.359111 | yes |
| Spcs1 | | | 147.082 | | 112.939 | -0.38108 | yes |
| Spock2 | | | 89.9439 | | 105.137 | 0.22517 | yes |
| Sptb | | | 7.95545 | | 10.1506 | 0.351544 | yes |
| Sptbn2 | | | 124.943 | | 157.719 | 0.336091 | yes |
| Srgap3 | | | 27.5427 | | 34.0272 | 0.305018 | yes |
| Srp14 | | | 99.7161 | | 81.8592 | -0.28468 | yes |
| Ssr4 | | | 55.9284 | | 42.3364 | -0.40168 | yes |
| Stmn1 | | | 671.141 | | 558.369 | -0.2654 | yes |
| Stmn2 | | | 236.88 | | 203.297 | -0.22057 | yes |
| Stmn3 | | | 614.808 | | 481.928 | -0.35132 | yes |
| Sub1 | | | 164.339 | | 130.623 | -0.33127 | yes |
| Suclg1 | | | 68.4544 | | 57.848 | -0.24288 | yes |
| Sumo1 | | | 83.7104 | | 65.8555 | -0.3461 | yes |
| Tacc1 | | | 18.8555 | | 22.9163 | 0.281395 | yes |
| Taldo1 | | | 66.97 | | 55.7093 | -0.2656 | yes |
| Tceal1 | | | 12.6865 | | 10.3433 | -0.29461 | yes |
| Tceb2 | | | 388.27 | | 270.504 | -0.52141 | yes |
| Tet2 | | | 0.717445 | | 1.05026 | 0.549811 | yes |
| Tgfbr3 | | | 1.40743 | | 1.93263 | 0.457506 | yes |
| Them4 | | | 13.182 | | 10.3607 | -0.34744 | yes |
| Timm13 | | | 53.7977 | | 43.6748 | -0.30075 | yes |
| Timm8b | | | 183.142 | | 136.771 | -0.4212 | yes |
| Tln2 | | | 7.00014 | | 8.65497 | 0.306145 | yes |
| Tma7 | | | 188.266 | | 135.277 | -0.47685 | yes |
| Tmco1 | | | 9.56261 | | 7.90166 | -0.27525 | yes |
| Tmem132d | | | 5.04208 | | 6.37609 | 0.338652 | yes |
| Tmem158 | | | 40.2779 | | 25.499 | -0.65955 | yes |
| Tmem242 | | | 57.7371 | | 46.8247 | -0.30223 | yes |
| Tmem245 | | | 4.10912 | | 5.05737 | 0.299556 | Yes |
| Tmem256 | | | 129.975 | | 99.657 | -0.38319 | Yes |
| Tmem258 | | | 49.1462 | | 32.5935 | -0.5925 | Yes |
| Tmem59 | | | 127.303 | | 101.144 | -0.33185 | Yes |
| Tmem60 | | | 16.7094 | | 12.4446 | -0.42513 | yes |
| Tmsb10 | | | 147.337 | | 121.785 | -0.27478 | Yes |
| Tmsb4x | | | 1010.09 | | 796.748 | -0.3423 | Yes |
| Tnpo2 | | | 28.5884 | | 33.8036 | 0.241745 | Yes |
| Tnr | | | 3.31642 | | 5.88957 | 0.828538 | Yes |
| Tnrc6b | | | 2.02586 | | 2.7214 | 0.425815 | Yes |
| Tomm5 | | | 54.1037 | | 41.6424 | -0.37767 | Yes |
| Tonsl | | | 93.8748 | | 78.7871 | -0.25278 | Yes |
| Tril | | | 8.06926 | | 10.2601 | 0.34654 | Yes |
| Txn1 | | | 72.839 | | 54.8116 | -0.41023 | yes |
| Txndc9 | | | 10.1598 | | 8.13472 | -0.32071 | yes |
| Txnl1 | | | 29.7281 | | 23.3994 | -0.34536 | yes |
| Ube2a | | | 29.2406 | | 23.4167 | -0.32044 | yes |
| Ubl5 | | | 350.326 | | 264.258 | -0.40676 | yes |
| Ublcp1 | | | 28.425 | | 21.3134 | -0.41539 | yes |
| Uchl1 | | | 589.001 | | 464.898 | -0.34135 | yes |
| Uchl3 | | | 26.1649 | | 20.0521 | -0.38388 | yes |
| Uqcc2 | | | 188.846 | | 145.598 | -0.37522 | yes |
| Uqcr10 | | | 330.075 | | 273.12 | -0.27326 | yes |
| Uqcr11 | | | 348.725 | | 281.491 | -0.309 | yes |
| Uqcrc2 | | | 140.268 | | 114.036 | -0.2987 | yes |
| Uqcrh | | | 395.88 | | 305.901 | -0.372 | yes |
| Uqcrq | | | 300.919 | | 201.276 | -0.5802 | yes |
| Usmg5 | | | 556.964 | | 379.611 | -0.55306 | yes |
| Usp31 | | | 8.65244 | | 10.3563 | 0.25933 | yes |
| Vapa | | | 102.798 | | 87.1073 | -0.23894 | yes |
| Vash1 | | | 4.79432 | | 5.81612 | 0.278732 | yes |
| Vbp1 | | | 31.5512 | | 25.2918 | -0.31902 | yes |
| Vdac3 | | | 81.0647 | | 65.8089 | -0.30079 | yes |
| Vsnl1 | | | 443.993 | | 364.254 | -0.2856 | yes |
| Ypel4 | | | 24.4174 | | 19.7179 | -0.30841 | yes |
| Zcchc18 | | | 111.62 | | 93.2065 | -0.26009 | yes |
| Zfp106 | | | 25.1978 | | 31.2749 | 0.311706 | yes |
| Zfp771 | | | 25.1294 | | 32.1017 | 0.353272 | yes |
| Zmiz2 | | | 52.9522 | | 64.2159 | 0.278241 | yes |
| Znfx1 | | | 9.4986 | | 11.9084 | 0.32619 | yes |
| Adcy1 | | | 48.6301 | | 61.0127 | 0.327262 | yes |
| Ago2 | | | 2.87286 | | 3.49297 | 0.281968 | yes |
| Alkbh5 | | | 6.82429 | | 8.6951 | 0.349523 | yes |
| Ankrd52 | | | 4.14704 | | 5.3991 | 0.38064 | yes |
| Atp1a2 | | | 183.871 | | 227.262 | 0.305663 | yes |
| Bcr | | | 11.7997 | | 14.4169 | 0.289006 | yes |
| Btg2 | | | 3.58469 | | 5.04202 | 0.492155 | yes |
| Cdc42bpb | | | 19.9957 | | 23.8211 | 0.25255 | yes |
| Chst2 | | | 18.7978 | | 22.2545 | 0.243536 | yes |
| Cmtm4 | | | 14.3859 | | 17.4019 | 0.274589 | yes |
| Crtc1 | | | 27.1903 | | 32.9383 | 0.276674 | yes |
| Dio2 | | | 21.771 | | 26.5589 | 0.286786 | yes |
| Dtl | | | 2.44313 | | 1.72614 | -0.50118 | yes |
| Dusp1 | | | 6.12652 | | 10.3275 | 0.753348 | yes |
| Fbxl18 | | | 2.89077 | | 4.34955 | 0.589411 | yes |
| Fosl2 | | | 2.43769 | | 3.38974 | 0.475657 | yes |
| Gatsl2 | | | 11.8953 | | 14.326 | 0.268244 | yes |
| Gfod1 | | | 13.5304 | | 17.0471 | 0.333328 | yes |
| Gm15800 | | | 8.3915 | | 10.9594 | 0.385171 | yes |
| Gm26917,Yam1 | | | 561.392 | | 835.354 | 0.57338 | yes |
| Gstm1 | | | 116.589 | | 136.152 | 0.223789 | yes |
| Hba-a1 | | | 93.9095 | | 118.536 | 0.33598 | yes |
| Hba-a2 | | | 120.914 | | 146.48 | 0.27673 | yes |
| Homer2 | | | 6.08544 | | 7.53581 | 0.308401 | yes |
| Igf1r | | | 2.74434 | | 3.45474 | 0.332121 | yes |
| Igsf9b | | | 1.65157 | | 2.21945 | 0.426365 | yes |
| Irf2bp2 | | | 5.15593 | | 6.579 | 0.351636 | yes |
| Klf13 | | | 18.0759 | | 24.2464 | 0.423703 | yes |
| Lars2 | | | 1435.57 | | 821.714 | -0.80491 | yes |
| Lix1l | | | 6.60878 | | 8.22638 | 0.315873 | yes |
| Lrrc58 | | | 8.03872 | | 11.3422 | 0.496657 | yes |
| Lzts1 | | | 14.6926 | | 18.0478 | 0.296728 | yes |
| Map1a | | | 92.5659 | | 117.052 | 0.338592 | yes |
| Mast3 | | | 40.9158 | | 48.7613 | 0.25308 | yes |
| Mir6236 | | | 69776.3 | | 34959.3 | -0.99706 | yes |
| Mir6236 | | | 1.61564 | | 7.45184 | 2.2055 | yes |
| Mn1 | | | 3.88176 | | 5.15909 | 0.410406 | yes |
| Mt3 | | | 442.807 | | 538.434 | 0.28209 | yes |
| Myh9 | | | 6.32096 | | 8.06921 | 0.352284 | yes |
| Ncan | | | 22.9592 | | 29.6578 | 0.369342 | yes |
| Ncs1 | | | 56.8467 | | 66.6845 | 0.230273 | yes |
| Npas4 | | | 1.43753 | | 2.14749 | 0.579058 | yes |
| Palm | | | 62.8705 | | 74.6174 | 0.247129 | yes |
| Paqr8 | | | 20.265 | | 25.3769 | 0.324525 | yes |
| Pdxk | | | 20.4812 | | 24.0069 | 0.229149 | yes |
| Plxnd1 | | | 12.1552 | | 14.4985 | 0.254333 | yes |
| Pnmal2 | | | 53.7835 | | 64.4608 | 0.261256 | yes |
| Pom121 | | | 7.66729 | | 9.23082 | 0.267741 | yes |
| Ppp1r16b | | | 14.7659 | | 19.1206 | 0.372856 | yes |
| Prr12 | | | 4.78392 | | 6.24815 | 0.385236 | yes |
| Ptrf | | | 6.70487 | | 9.75419 | 0.540812 | yes |
| Pvrl1 | | | 3.68628 | | 4.7094 | 0.353377 | yes |
| Rims4 | | | 16.1885 | | 19.5317 | 0.270846 | yes |
| Rps14 | | | 576.364 | | 484.466 | -0.25058 | yes |
| Rps21 | | | 961.324 | | 785.043 | -0.29225 | yes |
| Rps29 | | | 921.088 | | 775.335 | -0.24852 | yes |
| Sccpdh | | | 47.8915 | | 37.2021 | -0.36439 | yes |
| Scrt1 | | | 28.7186 | | 33.7821 | 0.234274 | yes |
| Sdc4 | | | 14.7008 | | 18.4531 | 0.327968 | yes |
| Sema3g | | | 1.03512 | | 1.53122 | 0.564882 | yes |
| Sft2d2 | | | 6.83864 | | 8.75494 | 0.356387 | yes |
| Shank2 | | | 17.4558 | | 21.2236 | 0.281962 | yes |
| Shank3 | | | 32.7673 | | 41.7592 | 0.349836 | yes |
| Sipa1l1 | | | 52.1639 | | 64.1818 | 0.299114 | yes |
| Snhg11 | | | 38.7457 | | 30.3723 | -0.35128 | yes |
| Socs7 | | | 13.3943 | | 16.323 | 0.28529 | yes |
| Spry2 | | | 12.4264 | | 16.5559 | 0.413934 | yes |
| Stox2 | | | 7.75961 | | 9.58476 | 0.304758 | yes |
| Synpo | | | 40.5177 | | 47.8544 | 0.240097 | yes |
| Thbd | | | 6.92168 | | 8.82913 | 0.351148 | yes |
| Tmcc2 | | | 43.692 | | 53.6485 | 0.296167 | yes |
| Tmem151b | | | 20.3138 | | 26.6033 | 0.389146 | yes |
| Traf3 | | | 4.56569 | | 6.15697 | 0.431387 | yes |
| Ttr | | | 0.28353 | | 6.53798 | 4.52723 | yes |
| Wfs1 | | | 35.7559 | | 42.5656 | 0.25151 | yes |
| Zranb2 | | | 45.9229 | | 37.6343 | -0.28717 | yes |
| Gm22405 | | | 41.0435 | | 161.86 | 1.97952 | yes |
| Arc | | | 6.45593 | | 21.5574 | 1.73948 | yes |
| Dusp6 | | | 13.7466 | | 17.604 | 0.356828 | yes |
| Egr1 | | | 22.5389 | | 38.3525 | 0.766907 | yes |
| Fos | | | 1.58833 | | 4.65726 | 1.55197 | Yes |
| Nr4a1 | | | 8.41942 | | 12.8925 | 0.614744 | Yes |
